# Photoferrotrophs Produce a PioAB Electron Conduit for Extracellular Electron Uptake

**DOI:** 10.1128/mBio.02668-19

**Published:** 2019-11-05

**Authors:** Dinesh Gupta, Molly C. Sutherland, Karthikeyan Rengasamy, J. Mark Meacham, Robert G. Kranz, Arpita Bose

**Affiliations:** a Department of Biology, Washington University in St. Louis, St. Louis, Missouri, USA; b Department of Mechanical Engineering and Materials Science, Washington University in St. Louis, St. Louis, Missouri, USA; c Institute of Materials Science and Engineering, Washington University in St. Louis, St. Louis, Missouri, USA; University of California, Berkeley

**Keywords:** photoferrotrophy, phototrophic EEU, *Rhodopseudomonas palustris* TIE-1, decaheme cytochrome *c*, Fe(II)-oxidation

## Abstract

Photoferrotrophy is a form of anoxygenic photosynthesis whereby bacteria utilize soluble or insoluble forms of ferrous iron as an electron donor to fix carbon dioxide using light energy. They can also use poised electrodes as their electron donor via phototrophic extracellular electron uptake (phototrophic EEU). The electron uptake mechanisms underlying these processes are not well understood. Using Rhodopseudomonas palustris TIE-1 as a model, we show that a single periplasmic decaheme cytochrome *c*, PioA, and an outer membrane porin, PioB, form a complex allowing extracellular electron uptake across the outer membrane from both soluble iron and poised electrodes. We observe that PioA undergoes postsecretory proteolysis of its N terminus to produce a shorter heme-attached PioA (holo-PioA_C_, where PioA_C_ represents the C terminus of PioA), which can exist both freely in the periplasm and in a complex with PioB. The extended N-terminal peptide controls heme attachment, and its processing is required to produce wild-type levels of holo-PioA_C_ and holo-PioA_C_B complex. It is also conserved in PioA homologs from other phototrophs. The presence of PioAB in these organisms correlate with their ability to perform photoferrotrophy and phototrophic EEU.

## Abstract (precis)

Some anoxygenic phototrophs use soluble iron, insoluble iron minerals (such as rust), or their proxies (poised electrodes) as electron donors for photosynthesis. However, the underlying electron uptake mechanisms are not well established. Here, we show that these phototrophs use a protein complex made of an outer membrane porin and a periplasmic decaheme cytochrome (electron transfer protein) to harvest electrons from both soluble iron and poised electrodes. This complex has two unique characteristics: (i) it lacks an extracellular cytochrome *c*, and (ii) the periplasmic decaheme cytochrome *c* undergoes proteolytic cleavage to produce a functional electron transfer protein. These characteristics are conserved in phototrophs harboring homologous proteins.

## INTRODUCTION

Several anoxygenic (nonoxygen evolving) phototrophs can grow by coupling oxidation of ferrous iron to carbon dioxide (CO_2_) fixation using the energy of light, a process called photoferrotrophy (1). Photoferrotrophy plays an important role in biogeochemical cycling of iron and impacts microbial ecology in marine and freshwater ecosystems (2–4). A phylogenetically diverse group of bacteria carry out this process in natural environments (5). Photoferrotrophs use both soluble ferrous iron [Fe(II)] and insoluble mixed-valence iron minerals as electron donors (6, 7). Photoferrotrophy is considered to be one of the most ancient types of photosynthesis and may represent a transition state between anoxygenic and modern oxygenic photosynthesis (8). In addition, photoferrotrophy is suggested as a primary metabolism responsible for early marine productivity (9) and a potential process responsible for the deposition of the archean banded iron formations (BIFs) (1, 10–13). Photoferrotrophs can also use electrons from a poised electrode to fix CO_2_ with light, a process called phototrophic extracellular electron uptake (EEU) (14, 15). Microbes capable of phototrophic EEU are good candidates for microbial electrosynthesis, a process in which microbes use electricity to produce biocommodities from CO_2_ (16–18). Despite continued interest in microbes capable of photoferrotrophy and phototrophic EEU, the mechanisms underlying these processes are poorly understood.

The bacterial outer envelope is nonconductive to electrons and is impermeable to insoluble iron minerals/electrodes (19, 20). Therefore, the ability of phototrophs to use the extracellular electron donors likely involves an extracellular electron uptake (EEU) process. Studies in the nonphototroph model bacterium Shewanella oneidensis suggest that the mechanism of electron transfer across the outer membrane (OM) employs a porin-cytochrome complex (21). The *Shewanella* complex is comprised of a porin (MtrB) and two decaheme cytochrome *c* proteins (MtrA and MtrC from periplasmic and extracellular sides, respectively) as an electron conduit. Deletion of the extracellular decaheme cytochrome *c* component of the conduit (MtrC and its paralogues, OmcA and MtrF) abrogates *Shewanella*’s ability to reduce Fe(III) or transfer electrons to electrodes (22–24). Recently, it has been shown that the *Shewanella* MtrCAB conduit may also be employed for electron uptake (electron transfer in the opposite direction from its native function) from a cathode to an intracellular reduction reaction (18, 25). In contrast to oxidation of insoluble iron/electrode, soluble iron oxidation could occur either extracellularly or in the periplasm of Gram-negative phototrophs (26–28). Evidence to date suggests that the oxidation of soluble iron in Rhodopseudomonas palustris TIE-1 (6) and *Rhodobacter* sp. strain SW2 (29) involves periplasmic iron oxidoreductases (PioA and FoxE, respectively). However, the oxidation of soluble iron could be extracellular because the product of this process is insoluble iron, which can be toxic if produced in the periplasm (10, 13, 30, 31). Genetic studies in R. palustris TIE-1, the only genetically tractable phototroph (32) that can perform both photoferrotrophy and phototrophic EEU (6, 7, 15), identified the *pioABC* operon as required for both of these processes (6, 15). The operon encodes PioA, a decaheme cytochrome *c* (cyt *c*), PioB, an outer membrane (OM) porin, and PioC, a high-potential iron-sulfur protein (HiPIP). A deletion mutant lacking *pioABC* cannot perform photoferrotrophy (6) and has a partial defect in phototrophic EEU (15). Beyond these genetic studies, only the iron-sulfur protein PioC has been biochemically characterized as a periplasmic electron transfer protein and proposed to shuttle electrons from PioA to the photosynthetic reaction center (33, 34). However, where Pio proteins oxidize Fe(II) during photoferrotrophy and how they transfer electrons across the outer membrane from poised electrodes during phototrophic EEU are unknown.

Here, we show that PioA undergoes novel postsecretory proteolysis of its N terminus to produce a decaheme-attached PioA (holo-PioA_C_, where PioA_C_ represents the C terminus of PioA). The holo-PioA_C_ is an iron oxidoreductase that forms a membrane-associated protein complex with PioB. The holo-PioA_C_B complex acts as an electron conduit in TIE-1 to accept electrons from the extracellular oxidation of both Fe(II) and from the poised electrodes (that mimic insoluble iron minerals). Importantly, this process employs a single decaheme cyt *c* (holo-PioA_C_) and a porin (PioB), with no apparent extracellular electron transfer protein. Therefore, the PioAB complex is distinct from other porin-cytochrome *c* systems found in nonphototrophic bacteria. The postsecretory proteolysis of the N-terminal extension of PioA-like homologs to produce a functional holo-PioA_C_ and the formation of holo-PioA_C_B electron conduit are conserved in phototrophs such as Rhodomicrobium vannielii and Rhodomicrobium udaipurense. Together, these results suggest that the PioAB system can serve as a molecular signature for photoferrotrophy and phototrophic EEU.

## RESULTS AND DISCUSSION

### Production and purification of recombinant PioA from Escherichia coli.

**FIG 1:**
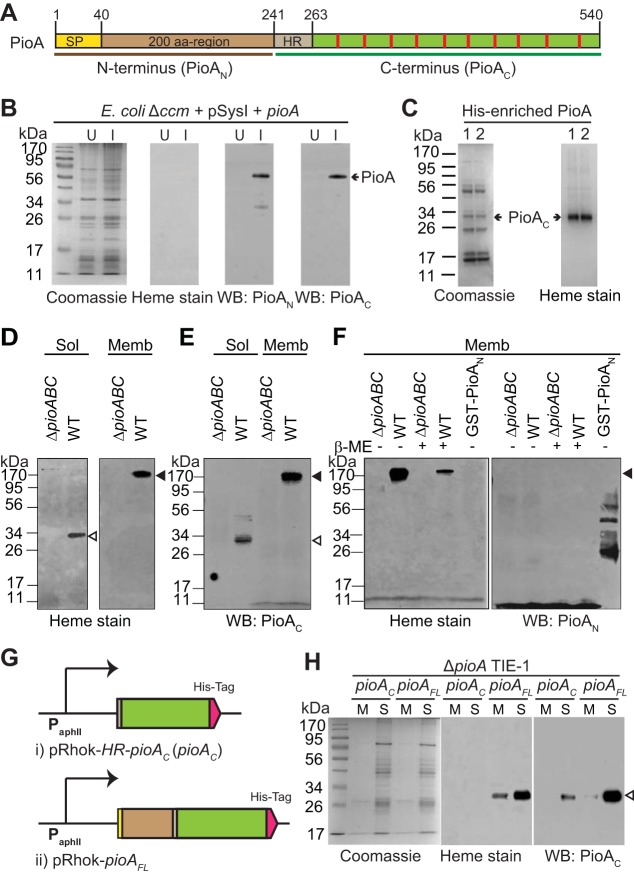
A 34-kDa holo-PioA_C_ is produced both in TIE-1 and E. coli. (A) Schematic diagram of the annotated PioA sequence representing N and C termini of PioA. SP, predicted Sec signal peptide; HR, hydrophobic region; red strip, a heme binding site. (B and C) PioA expression and analysis in E. coli. Results of Coomassie and heme staining and Western blotting (WB) with PioA-specific antibodies (anti-PioA_N_ and anti-PioA_C_) of total cell lysate of uninduced (U) and induced (I) cells are shown in panel B. Arrow, ∼54-kDa PioA. Results of Coomassie and heme staining of His-enriched PioA are shown in panel C. Arrow, heme-stainable PioA (∼34 kDa, PioA_C_). Lanes 1 and 2 represent two replicates. (D to H) PioA analysis in TIE-1. Heme staining (D) and immunoblotting with anti-PioA_C_ antibodies (E) of the soluble (Sol) and membrane (Memb) fractions from wild type (WT) and Δ*pioABC* mutant are shown. Mass spectrometry analysis of the >170-kDa (membrane fraction) PioA bands identified it as PioAB complex (Fig. S4A). Heme staining and immunoblotting with anti-PioA_N_ antibodies for the membrane fractions of WT and Δ*pioABC* mutant are shown in panel F. Treatment with β-mercaptoethanol (β-ME) to help unfold the PioAB complex is indicated. An affinity-purified glutathione *S*-transferase (GST)-PioA_N_ fusion protein was used as a positive control. Schematics of the constructs expressing either only the C terminus of PioA (HR-PioA_C_) (i) or a full-length PioA (PioA_FL_) with a C-terminal His tag under P*_aphII_*, a constitutive promoter (ii) are shown in panel G. Coomassie and heme staining and immunoblotting with anti-PioA_C_ antibodies on His-enriched PioA from membrane (M) and soluble (S) fractions of a Δ*pioA* TIE-1 mutant expressing either HR-PioA_C_ or PioA_FL_ are shown in panel H. A Coomassie gel was run to ensure nearly equal protein loading. Open and filled triangles indicate holo-PioA_C_ (∼34 kDa) and holo-PioA_C_B complex (>170 kDa), respectively.

The *pioA* gene (Rpal_0817) encodes a 540-amino-acid (aa) decaheme cyt *c* with a canonical signal peptide (SP; aa 1 to 40), a 200-residue N terminus, and the C terminus (Fig. 1A). The C terminus contains a hydrophobic region (HR; aa 241 to 263) followed by 10 sites for *c*-type heme (CXXCH) attachment (Fig. 1A). PioA has a large (∼200 aa) N terminal region not present in previously characterized decaheme cyt *c* homologs such as MtrA and MtoA (see Fig. S1 in the supplemental material). This N-terminal extension is conserved in PioA homologs annotated in the genomes of different phototrophic bacteria and does not harbor any clear protein domains (Fig. S2). Previous translational fusion studies suggest that a full-length PioA with its N terminus is produced in TIE-1 (35). Here, we wanted to investigate the role of the N terminal extension in these decaheme homologs by studying PioA. Although PioA expression in TIE-1 is upregulated only under photoferrotrophy, its slow growth (doubling time of 80 ± 10 h) and low biomass yields under this condition make it difficult to purify sufficient protein for biochemical analysis.

10.1128/mBio.02668-19.1 FIG S1 PioA has an N-terminal extension compared to the lengths of previously characterized decaheme homologs, MtrA and MtoA. Larger N terminus of PioA is highlighted. Predicted Sec signal peptides (processed and not present in the final protein) for these proteins were excluded in this alignment. Download FIG S1, PDF file, 0.8 MB. Copyright © 2019 Gupta et al. 2019 Gupta et al. This content is distributed under the terms of the Creative Commons Attribution 4.0 International license.

10.1128/mBio.02668-19.2 FIG S2 Decaheme cyt *c* homologs with larger N termini (PioA-like) are an exclusive trait of phototrophic bacteria. (A) PioA-like homologs are encoded by operons that have *pioABC* genes in phototrophs (red box). Gene ortholog neighborhood regions with *pioA* (Rpal_0817) gene’s bidirectional best hits are shown (jgi.doe.gov) (Chen et al., Nucleic Acids Res 47:D666–D677, 2019, https://doi.org/10.1093/nar/gky901). The *pioA* homologs are shown in red; *pioB* homologs and *pioC* homologs are shown in light yellow. Genes of the same color (except light yellow) represent the same orthologous group. Asterisk represents a joining point of contigs JFZJ010000142 and JFZJ010000052 in the R. udaipurense JA643 genome. Names of phototrophs are represented in black and Shewanella oneidensis MR-1 (heterotroph) and Sideroxydans lithotrophicus ES-1 (chemolithotroph) are represented in red. (B) PioA-like homologs contain larger N terminus and internal HR. PioA-like (black) and MtrA-like (red) decaheme homologs are shown. Internal HR regions are highlighted in yellow. Rpal, R. palustris; Rvan, R. vannielii; Ruda, R. udaipurense; Sone, Shewanella oneindensis; and Slit, Sideroxydans lithotrophicus. Download FIG S2, PDF file, 1.4 MB. Copyright © 2019 Gupta et al. 2019 Gupta et al. This content is distributed under the terms of the Creative Commons Attribution 4.0 International license.

10.1128/mBio.02668-19.4 FIG S4 Characterization of PioAB complex. (A) Mass spectrometry result of PioAB complex (>170 kDa heme-stainable band). Peptides detected by mass spectrometry analysis are highlighted in the PioA (top) and PioB (bottom) sequences. (B) Holo-PioA_C_B complex shows heat modifiability property. Coomassie and heme staining of the membrane fraction with/without heat treatment from Δ*pioABC* mutant and WT TIE-1. Heme-stainable band at ∼120 kDa (lane 2) shifts to >170 kDa (lane 4) after heat treatment. Porins, after heating, form an excessive network of hydrogen bonding that holds β-strands together into the β-barrel shape, resulting in an SDS-resistant structure responsible for the shift in SDS-PAGE. The heat modifiability of holo-PioA_C_B complex indicates that PioB is in its folded β-barrel state (N. Noinaj, A. J. Kuszak, and S. K. Buchanan, Methods Mol Biol 1329:51–56, 2015, https://doi.org/10.1007/978-1-4939-2871-2_4). Download FIG S4, PDF file, 1.1 MB. Copyright © 2019 Gupta et al. 2019 Gupta et al. This content is distributed under the terms of the Creative Commons Attribution 4.0 International license.

We heterologously expressed PioA with a C-terminal His tag in the E. coli (Δ*ccm*) RK103 strain carrying the *ccmABCDEFGH* genes under an inducible promoter to ensure heme attachment (36). For correct localization of the protein in E. coli’s periplasm, we replaced the PioA signal peptide with a validated signal peptide of the cytochrome *c_4_* gene (36). We confirmed production of the predicted full-length apo-PioA (∼54 kDa) in total E. coli lysate by immunoblotting with antibodies specific to the N-terminal 200-aa region (anti-PioA_N_) and the C terminus (anti-PioA_C_) of PioA (Fig. 1B). However, an ∼34-kDa heme-containing polypeptide was observed upon enrichment using hexahistidine affinity purification (Fig. 1C). Heme stains were used to detect covalently bound (*c*-type) heme in the protein (37). The polypeptide was immunodetected with anti-PioA_C_ but not with anti-PioA_N_ antibodies (Fig. S3A). These results suggest that the 34-kDa protein is PioA after removal of the N-terminal 200-aa region. Here, the recombinant holo-PioA is termed holo-PioA_C_^r^.

10.1128/mBio.02668-19.3 FIG S3 Production and characterization of recombinant holo-PioA_C_^r^. (A) Coomassie and heme staining and Western blots (WB) with PioA-specific antibodies of His-enriched PioA protein samples. PioA-1 and PioA-2 are replicates. (B) UV-visible light spectral analysis of the affinity-purified holo-PioA_C_^r^ under aerobic conditions. The spectra for holo-PioA_C_^r^ are typical of *c*-type cytochromes with absorption maxima at 408 nm (γ) and 540 nm in the oxidized state (black) and 418 nm (γ), 523 nm (β), and 551 nm (α) in the reduced state (red). Download FIG S3, PDF file, 0.9 MB. Copyright © 2019 Gupta et al. 2019 Gupta et al. This content is distributed under the terms of the Creative Commons Attribution 4.0 International license.

### TIE-1 produces a 34-kDa holo-PioA_C_ that exists as a free periplasmic protein and in a complex with PioB.

To investigate the biological relevance of PioA N-terminal processing in the native photosynthetic host, we isolated soluble and membrane fractions of photoautotrophically grown wild-type (WT) and Δ*pioABC* mutant strains of TIE-1. We observed two heme-containing polypeptides: an ∼34-kDa band in the soluble fraction and a larger (>170-kDa) band in the membrane fraction of WT TIE-1 (Fig. 1D). Both bands reacted with anti-PioA_C_ antibodies (Fig. 1E) but not with anti-PioA_N_ antibodies (Fig. 1F). Mass spectrometry analysis of these bands identified only the C-terminal peptides of PioA, consistent with processing of the N-terminal region in these heme-attached forms of PioA. These results suggest that TIE-1 produces only the 34-kDa heme-attached C terminus of PioA (here termed holo-PioA_C_). Mass spectrometry analysis of the >170-kDa protein band identified it as PioA_C_B complex (Fig. S4A). This band also demonstrates heat modifiability (Fig. S4B), a feature reported for bacterial porins (38–42). Indeed, the holo-PioA_C_B migrates as an ∼120-kDa band without heat treatment and shifts to >170 kDa when the sample is heated to 90°C for 3 min (Fig. S4B). The 120-kDa holo-PioA_C_B band (unheated) likely represents the observed size of holo-PioA_C_ (∼34 kDa) plus the expected size of PioB (∼87 kDa), further indicating that the complex is composed of holo-PioA_C_ (that lacks the N-terminal region) and PioB. Overall, these results suggest that TIE-1 produces a 34-kDa holo-PioA_C_ that exists in two forms, a free soluble periplasmic protein and a membrane-associated protein in complex with PioB (holo-PioA_C_B).

Holo-PioA_C_ produced natively in TIE-1 is the same size as holo-PioA_C_^r^ produced heterologously in E. coli (as determined by SDS-PAGE). A similarly sized heme-stainable PioA (∼40 kDa) was previously observed from the soluble fraction of TIE-1 by Jiao and Newman (6). The size observed for holo-PioA_C_ is comparable to the size of the previously characterized decaheme homologs MtrA and MtoA (21, 31). Our biochemical results suggest that holo-PioA_C_ is the functional component of a PioA_C_B complex in TIE-1. However, holo-PioA_C_ lacks the N-terminal 200-aa region of the predicted PioA protein. The question remains as to how TIE-1 produces holo-PioA_C_. There are two possibilities: (i) full-length PioA is produced and processed to produce holo-PioA_C_, or (ii) an internal methionine closer to the first heme attachment site is the functional start codon. Interestingly, the internal hydrophobic region (HR) of the C terminus of PioA starts with a methionine residue (Met241), and Met241 is the only internal methionine from which a putative Sec signal peptide is predicted based on PredTat (43). It is therefore possible that Met241 is used as a start codon for PioA, with the Sec signal in the HR serving as a periplasmic signal peptide and with the resulting protein being ∼34 kDa. There is, however, no observable canonical ribosome binding site (RBS) upstream of Met241. To directly test this possibility, we expressed the *pioA* gene encoding only the C terminus of PioA from the HR (241 to 540 aa; HR-PioA_C_) with a C-terminal 6×His tag in the Δ*pioA* mutant using a pRhokS-2 plasmid under a constitutive promoter, P*aphII*, along with a strong RBS (Fig. 1G) (44). We observed (by PioA_C_ Western blotting [WB]) the expected size of HR-PioA_C_ upon enrichment using hexahistidine affinity purification, suggesting that this version of the protein can be produced artificially in TIE-1 (Fig. 1H, WB:PioA_C_). However, HR-PioA_C_ was not heme attached as determined by heme staining (Fig. 1H, PioA_C_). Because *c*-heme maturation will depend on the periplasmic transport of HR-PioA_C_, the above result suggests that the Sec signal within the HR is not a functional periplasmic signal and that Met241 does not serve as an alternative start codon for PioA. These results demonstrate the importance of the N terminus (signal peptide and 200-aa region) in the synthesis of holo-PioA_C_ in TIE-1.

### N-terminal processing of PioA is required for photoferrotrophy.

**FIG 2:**
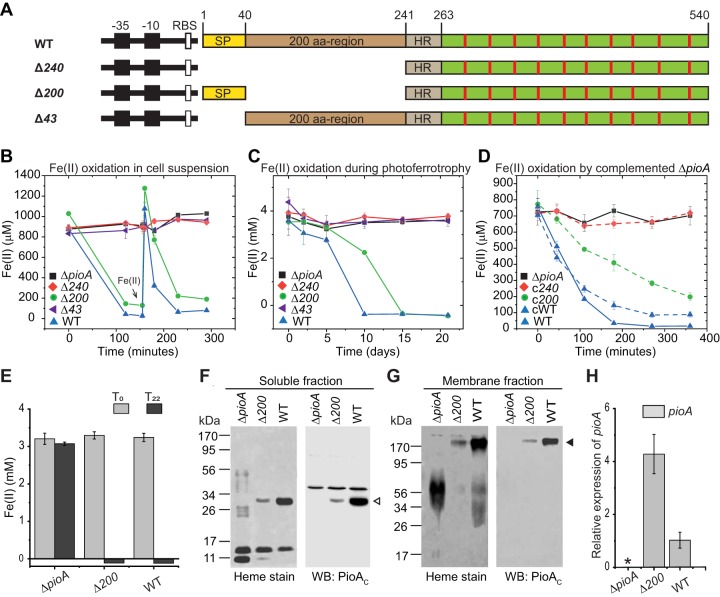
Production of the holo-PioA_C_ (34 kDa) is required for photoferrotrophy in TIE-1. (A) Schematic representation of the *pioA* gene in the wild-type (WT) TIE-1 and Δ240, Δ200, and Δ43 TIE-1 mutant genomes. The mutants lack regions encoding the N terminus (Δ240), the 200-aa region (Δ200), and the putative signal peptide (Δ43) of PioA protein. (B) Fe(II) oxidation by WT and TIE-1 mutants in cell suspension assays. Data shown are representative of three independent experiments. Error bars are means ± standard deviations of three technical replicates. (C) Fe(II) oxidation by WT and TIE-1 mutants during photoferrotrophic growth. Data are means ± standard deviations of three biological replicates. Cell growth during photoferrotrophy was determined by OD_660_ measurements at indicated time points and protein quantification at the initial (*T*_0_) and final (*T_f_*) time points (Fig. S5). (D) Complementation of the Fe(II)-oxidizing ability of Δ*pioA* mutant by expressing *pioA*-containing plasmids that mimic the Δ240, Δ200, and WT genotypes. Data shown are representative of three independent experiments. Error bars are means ± standard deviations of three technical replicates. (E) Fe(II) oxidation by the Δ*pioA* and Δ200 mutants and WT TIE-1. Fe(II) concentration at the initial time (*T*_0_) and after 22 h (*T*_22_) of Fe(II) exposure are shown. Error bars are means ± standard deviations of three technical replicates. (F and G) Heme staining and Western blotting (WB) with anti-PioA_C_ antibodies for the soluble (F) and membrane (G) fractions of cells harvested after 22 h. Upregulation of other cyt *c* proteins was observed for the *pio* mutants compared to expression in the WT. Open and filled triangles indicate holo-PioA_C_ and holo-PioA_C_B complex, respectively. (H) Relative expression of *pioA* (normalized to that of *recA*) in cells harvested after 22 h. qRT-PCR data are means ± standard errors for three biological replicates assayed in duplicate. The asterisk represents a low value (0.01) for the Δ*pioA* mutant control.

To gain insights into the biological role of N-terminal processing, we engineered a series of N-terminal-PioA chromosomal deletion mutants in TIE-1 (Fig. 2A). We tested the ability of these mutants to perform Fe(II) oxidation. The Δ240 mutant lacks the complete N terminal region that includes both the predicted signal peptide and the 200-aa region, the Δ43 mutant lacks only the signal peptide, and the Δ200 mutant lacks only the 200-aa region but contains the intact signal peptide. Photoautotrophically grown TIE-1 cells with hydrogen were used for cell suspension assays. We observed that the Δ200 mutant oxidized Fe(II) both in the cell suspension assays and during photoferrotrophy (Fig. 2B and C, green line, and Fig. S5). In contrast, the Δ240 and Δ43 mutants, which lack the signal peptide that is essential for periplasmic export, were unable to oxidize Fe(II) or perform photoferrotrophy (Fig. 2B and C, red and purple lines, respectively). Furthermore, only the Δ200 construct rescued the phenotype of a Δ*pioA* mutant when expressed in *trans* from a plasmid (Fig. 2D). The ability of the Δ200 mutant to oxidize Fe(II) suggests that holo-PioA_C_ is a functional iron oxidase in TIE-1. It also demonstrates that the N-terminal 200-aa region of PioA is not directly involved in the ability of PioA to oxidize Fe(II).

10.1128/mBio.02668-19.5 FIG S5 Δ200 mutant can grow via photoferrotrophy. Photoferrotrophic growth of WT TIE-1 and Δ*pioA*, Δ240, Δ200, and Δ43 mutants was determined by OD_660_ measurement (A) and total protein quantification at the initial (*T*_0_) and final (*T_f_*) time points during photoferrotrophy (B). Data shown in panels A and B are means ± standard deviations of three biological replicates. The *P* values were determined by Student’s *t* test (1 star, *P* < 0.05; 2 stars, *P* < 0.01; 3 stars, *P* < 0.001). Download FIG S5, PDF file, 0.8 MB. Copyright © 2019 Gupta et al. 2019 Gupta et al. This content is distributed under the terms of the Creative Commons Attribution 4.0 International license.

**FIG 3:**
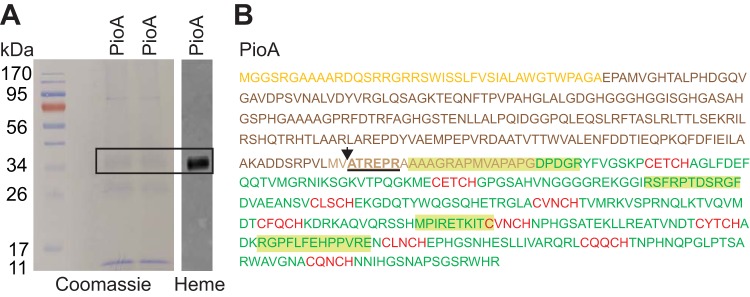
PioA is processed at Ala243 in the hydrophobic region. (A) Coomassie and heme staining of affinity-purified holo-PioA_C_ from the Δ*pioA* TIE-1 mutant expressing full-length *pioA* under the *pio* promoter with a C-terminal His tag. The band corresponding to the heme band (34 kDa, boxed) from the Coomassie blot was used for Edman degradation analysis (5-aa N-terminal sequencing). (B) PioA sequence showing N-terminal amino acids determined in Edman analysis of 34-kDa holo-PioA_C_ (ATREPR, underlined). The arrowhead indicates a cleavage site between Val242 and Ala243. Colors of peptides are consistent with the colors of different regions (Sec signal, 200-aa, HR, and heme-binding C terminus) of PioA represented in the models in Fig. 1A and 2A. The peptides detected by mass spectrometry analysis of the 34-kDa PioA band are highlighted.

Although the Δ200 mutant was able to grow via photoferrotrophy, it had an extended lag phase compared to that of the WT (Fig. 2C). We examined whether this lag is due to the difference in PioA concentrations between these two strains. To approximate photoferrotrophy and obtain sufficient cells for total protein extraction, strains grown photoautotrophically with hydrogen (optical density at 660 nm [OD_660,_] of 1.0) were exposed to 3 mM Fe(II) and harvested after 22 h when all Fe(II) was oxidized (Fig. 2E). We observed a smaller amount of both free holo-PioA_C_ and holo-PioA_C_B complex in the Δ200 mutant than in the WT (Fig. 2F and G). We also assessed the relative transcript levels of *pioA* under these conditions and observed a slight increase in *pioA* transcripts in the Δ200 mutant compared to the level in the WT (Fig. 2H). This result rules out the possibility that the lower level of free holo-PioA_C_ and holo-PioA_C_B complex in the Δ200 mutant could be due to lower protein expression in the mutant. Therefore, we propose that the N-terminal 200-aa region of PioA plays a role in maintaining the concentration of holo-PioA_C_ in TIE-1 at wild-type levels. Edman degradation analyses of holo-PioA_C_ purified from TIE-1 identified Ala243 as the N-terminal residue of the processed protein (Fig. 3). Cleavage at this site would produce a protein with a predicted molecular weight of 34 kDa, as calculated for the holo-PioA_C_.

### N-terminally processed PioA is an iron oxidase.

**FIG 4:**
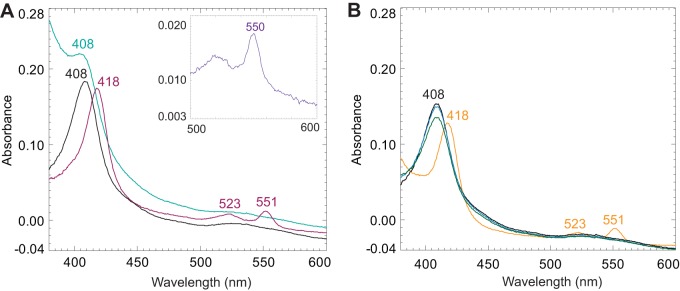
The holo-PioA_C_ is an iron oxidoreductase. UV-visible light spectral analysis of the affinity-purified PioA_C_^r^ was performed under anaerobic conditions. (A) Purified PioA (black), PioA reduced with sodium dithionite (purple), and reoxidized PioA (teal blue) after addition of Fe(III) chloride solution (100 μM) to reduced-PioA. The inset shows a pyridine hemochrome assay. (B) Purified PioA (black), PioA after addition of Fe(II) chloride solution (blue), PioA after increasing the pH to ∼8 (dark green), and PioA after increasing pH to ∼9 to 10 (orange).

The fact that holo-PioA_C_ is required for Fe(II) oxidation in TIE-1 suggests that it is an iron oxidoreductase. Thus, we affinity purified holo-PioA_C_^r^ and assayed its iron oxidation capacity by UV-visible light (UV-Vis) spectroscopy under anaerobic conditions. The UV-Vis spectra of holo-PioA_C_^r^ show the spectral signatures of typical *c*-type cytochromes (Fig. 4A and Fig. S3B). Covalently bound heme in holo-PioA_C_^r^ was determined by observing a 550-nm α peak in the pyridine hemochrome assay (Fig. 4A, inset) (45, 46). The dithionite-reduced holo-PioA_C_^r^ (Fig. 4A, purple) was reoxidized after addition of Fe(III) chloride solution, as indicated by a shift of the Soret peak back from 418 to 408 nm and disappearance of the α and β peaks at 551 and 523 nm, respectively (Fig. 4A, teal blue). This spectral characteristic of holo-PioA_C_^r^ is similar to that of the reduced-MtrCAB complex after addition of Fe(III) citrate (21). As reported for the decaheme cyt *c* MtoA (31), the reduction of holo-PioA_C_^r^ with Fe(II) chloride was pH dependent and only detected at a basic pH of 9 to 10 (Fig. 4B). These results demonstrate that holo-PioA_C_^r^ can donate electrons to Fe(III) and accept electrons from Fe(II). Thus, the N-terminal 200-aa region of PioA is not required for iron oxidase activity, but its processing is required for proper attachment of the heme groups to PioA, suggesting a regulatory role during PioA maturation.

### The holo-PioA_C_B complex catalyzes the extracellular oxidation of iron *in vivo*.

**FIG 5:**
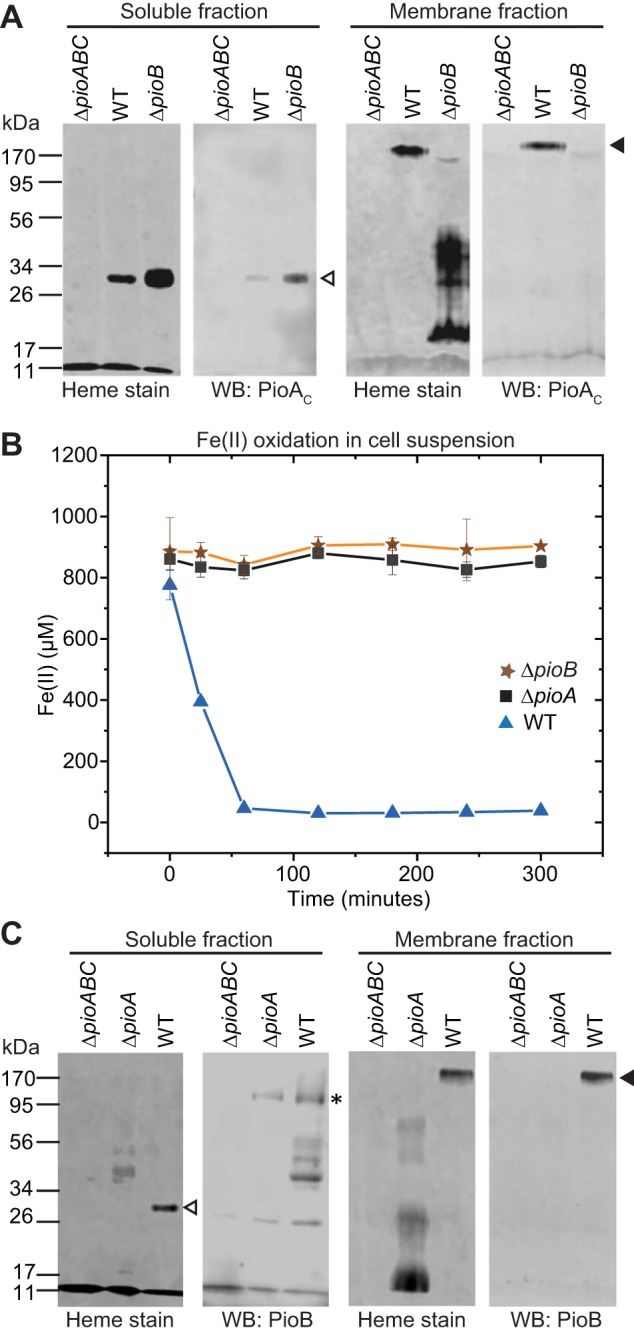
PioA and PioB codependence for outer membrane incorporation. (A) Heme staining and anti-PioA_C_ Western blotting (WB) of proteins in the soluble and membrane fractions of the Δ*pioABC* mutant, WT, and Δ*pioB* mutant. Open and filled triangles indicate holo-PioA_C_ and holo-PioA_C_B complex, respectively. (B) Fe(II) oxidation by the WT and the Δ*pioABC* and Δ*pioB* mutants in cell suspension assays. Data shown are representative of three independent experiments. Error bars are means ± standard deviations of three technical replicates. (C) Heme staining and immunoblotting with anti-PioB antibodies for the soluble and membrane fractions of the Δ*pioABC*, Δ*pioA*, and WT strains. The asterisk indicates free PioB.

An important unknown aspect of photoferrotrophy is the location of the Fe(II) oxidation activity in the bacterial cell. Our results suggest that Fe(II) oxidation could occur either in the periplasm by soluble holo-PioA_C_ or extracellularly by the holo-PioA_C_B complex. Both activities could also occur simultaneously in TIE-1. To dissect the roles of soluble holo-PioA_C_ and holo-PioA_C_B complex, we used the TIE-1 Δ*pioB* mutant. Deletions of *pioA*, *pioB*, and *pioC* in TIE-1 have been shown to be nonpolar (6). First, we tested the fate of holo-PioA_C_ in the absence of the PioB protein. Analysis of holo-PioA_C_ in the Δ*pioB* mutant showed that the protein is present only in the soluble fraction and not in the membrane fraction (Fig. 5A). This result agrees with the bioinformatic prediction of PioA as a periplasmic soluble protein and reveals the importance of PioB in the localization of PioA to the membrane. Because the Δ*pioB* mutant contains only soluble holo-PioA_C_, it serves as an ideal construct to investigate the role of periplasmic soluble holo-PioA_C_ in Fe(II) oxidation. We found that Δ*pioB* cannot oxidize Fe(II) in cell suspension assays (Fig. 5B). Because periplasmic holo-PioA_C_ is produced in the Δ*pioB* mutant, this result indicates that Fe(II) oxidation is unlikely to be carried out by soluble holo-PioA_C_ in the periplasm. Another explanation for this result might be that PioB acts as an Fe(II) import porin that makes Fe(II) available to periplasmic PioA for iron oxidation and/or as an Fe(III) export porin to remove Fe(III) from the cell. This would prevent accumulation of iron oxides in the periplasm, likely a lethal event for the cell (6). However, based on precedence from the MtrAB system in *Shewanella* (21, 47), PioB might exist only as a PioAB complex in the outer membrane. Moreover, in the MtrAB system in *Shewanella*, MtrB requires MtrA to enter the outer membrane (OM), and, therefore, the two proteins are inserted into the OM as a MtrAB complex (21, 47). To this end, we investigated the fate of PioB in the absence of PioA by using a Δ*pioA* mutant. Similar to observations of MtrAB, using immunoblotting with an antibody specific to PioB, we observed the presence of PioB in the soluble fraction but not in the membrane fraction of the Δ*pioA* mutant (Fig. 5C). This result suggests that the holo-PioA_C_ and PioB localize together as a complex in the OM, and, therefore, PioB is likely not present as a discrete porin in TIE-1. This result is further supported by the fact that we could detect only the holo-PioA_C_B complex and not free PioB in the membrane fraction of WT TIE-1 (Fig. 5C). Together, these results suggest that it is unlikely that soluble holo-PioA_C_ performs periplasmic Fe(II) oxidation and supports the hypothesis that the holo-PioA_C_B complex catalyzes Fe(II) oxidation extracellularly.

### The holo-PioA_C_B complex is required for phototrophic EEU in TIE-1.

**FIG 6:**
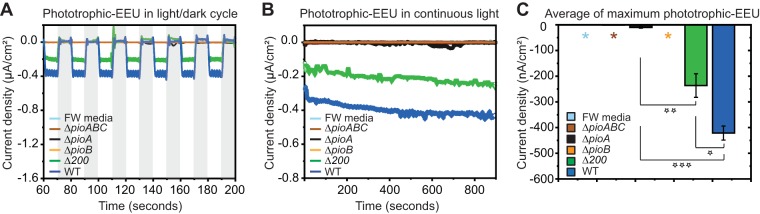
The holo-PioA_C_B complex is responsible for phototrophic EEU in TIE-1. Electron uptake (represented as current density) by the Δ*pioABC*, Δ*pioA*, Δ*pioB*, Δ200, and WT TIE-1 strains in the microfluidic bioelectrochemical cell (μ-BEC) under a light-dark cycle (shaded region) (A) and under continuous light (B) are shown. Data shown are representative of three experiments. (C) Average maximum electron uptake under continuous light at a time point of 600 s is represented as a bar diagram. Asterisks represent low current density values for a no-cell control (FW medium, 0.16 nA/cm^2^), Δ*pioABC* mutant (−1.31 nA/cm^2^), and the Δ*pioB* mutant (−0.45 nA/cm^2^). Error bars are means ± standard deviations of three biological replicates. The *P* values were determined by Student's *t* test (1 star, *P* < 0.05; 2 stars, *P* < 0.01; 3 stars, *P* < 0.001).

In addition to Fe(II), TIE-1 can oxidize insoluble iron minerals, such as hematite (7), and electrodes poised at the potential of insoluble iron minerals (15). Because these insoluble electron donors cannot cross the bacterial envelope, they are most likely oxidized by TIE-1 extracellularly via the holo-PioA_C_B complex. Poised electrodes in bulk reactors have been used as proxies of natural interactions between microbes and minerals such as iron oxides (15, 48, 49). However, in such bulk reactors different factors, such as extracellular enzymes (50), presence of planktonic cells, mediators, and abiotic reactions, could affect the electrochemical signals (15, 51). Therefore, we examined the involvement of the holo-PioA_C_B complex in EEU using a microfluidic bioelectrochemical cell (μ-BEC) developed in our laboratory (14). In the μ-BEC, an electrode poised at +100 mV versus a standard hydrogen electrode (SHE) was used to mimic the redox potential of insoluble iron minerals (15). We monitored and compared the light-dependent electron uptake ability of WT TIE-1 and mutants. Intermediate electron uptake by the Δ200 mutant was observed compared to that of the WT TIE-1 (Fig. 6A to C, green and blue, respectively; Table S1). This corroborates the observation that the Δ200 mutant contains a smaller amount of holo-PioA_C_B complex (Fig. 2G). We did not detect electron uptake by either the Δ*pioABC or* Δ*pioA* mutant. Similar to findings with respect to Fe(II) oxidation, Δ*pioB* mutant could not perform phototrophic EEU (Fig. 6A to C, light brown). This result shows that the holo-PioA_C_B complex, but not free periplasmic holo-PioA_C_, is responsible for phototrophic EEU in TIE-1. Phototrophic EEU has been studied in bulk bioelectrochemical systems (BESs) (15) and in μ-BECs (14). In BESs, both planktonic cells (70% electron uptake) and biofilm-attached cells (30% electron uptake) contribute to phototrophic EEU (14, 15). The μ-BEC collects data only from biofilm-attached cells. Therefore, our results from the μ-BEC suggest that in biofilm-attached cells, the PioABC system is essential for phototrophic EEU. This supports previous results from bulk BESs where the Δ*pioABC* mutant lost 30% electron uptake ability (15). Overall, our results indicate that the holo-PioA_C_B complex serves as an electron conduit in TIE-1 to take up electrons from poised electrodes that mimic insoluble iron minerals.

10.1128/mBio.02668-19.8 TABLE S1 Average maximum current uptake (nA cm^−2^) under continuous light in μ-BECs for different bacterial strains. Download Table S1, DOCX file, 0.01 MB. Copyright © 2019 Gupta et al. 2019 Gupta et al. This content is distributed under the terms of the Creative Commons Attribution 4.0 International license.

### Postsecretory processing of PioA and holo-PioA_C_B complex formation is a conserved trait in other phototrophs.

**FIG 7:**
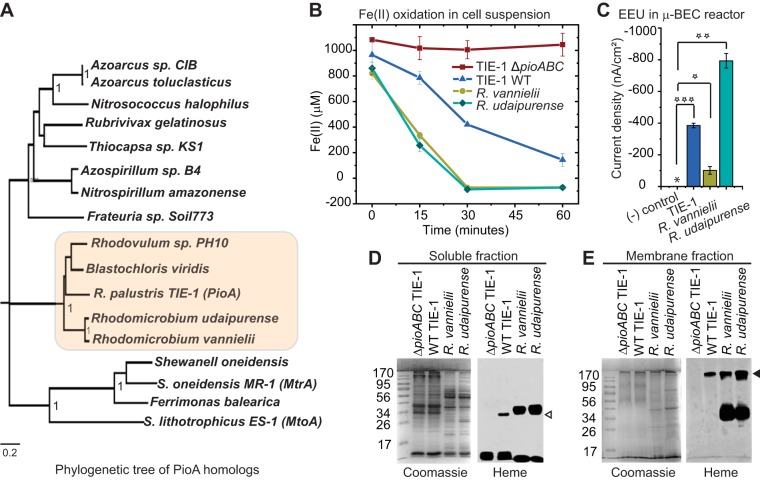
Phototrophic bacteria, Rhodomicrobium vannielii DSM 162 and R. udaipurense JA643, produce a PioAB complex and perform Fe(II) oxidation and phototrophic EEU. (A) Neighbor-joining phylogenetic tree of decaheme cyt *c* homologs of MtrA-like and PioA-like proteins. The highlighted clade contains a group of phototrophs that contains only PioA-like decaheme homologs. The clade clusters in the same way even when the N-terminal domain is excluded from the alignment. (B) Fe(II) oxidation by R. vannielii DSM 162 and R. udaipurense JA643 in cell suspension assays. Data shown are representative of three independent experiments. Error bars are means ± standard deviations of three technical replicates. Wild-type TIE-1 and the Δ*pioABC* mutant were used as the positive and negative controls, respectively. (C) Maximum phototrophic EEU (represented as current density) by R. vannielii DSM 162 and R. udaipurense JA643 in a microfluidic bioelectrochemical cell (μ-BEC) under continuous light. Asterisk represents a low average current density (0.0046 nA/cm^2^) for a no-cell negative (−) control. Error bars are means ± standard deviations of three biological replicates. The *P* values were determined by Student's *t* test (1 star, *P* < 0.05; 2 stars, *P* < 0.01; 3 stars, *P* < 0.001). (D and E) Coomassie and heme staining for the soluble and membrane fractions, as indicated, of H_2_CO_2_ grown TIE-1 Δ*pioABC*, WT TIE-1, R. vannielii DSM 162, and R. udaipurense JA643. Open and filled triangles indicate a smaller heme-stainable band (like holo-PioA_C_) in the soluble fraction and a higher-molecular-mass band of >170 kDa (like holo-PioA_C_B complex) in the membrane fraction, respectively. Mass spectrometry analysis of >170-kDa heme-stainable bands from R. vannielii DSM 162 and R. udaipurense JA643 confirm the presence of PioA and PioB homologs from the respective bacteria (see Fig. S6 in the supplemental material).

Homologs of PioA/MtrA and PioB/MtrB occur as operons in the genomes of many proteobacteria (15, 21). Sequence alignment of decaheme homologs separates them out into two groups, one without the N-terminal extension (MtrA-like) and other with the N-terminal extension (PioA-like) (Fig. S2B). Additionally, phylogenetic analysis clusters bacteria with PioA-like homologs in a distinct clade (Fig. 7A, highlighted clade). Interestingly, this clade includes phototrophic bacteria known to be photoferrotrophs such as TIE-1 (32) and Rhodomicrobium vannielii (52). Although the N-terminal extensions vary in length and are not conserved at the level of amino acid sequence, the presence of a larger N terminal region and an internal hydrophobic region (HR) upstream of the first heme-binding motif are conserved features of PioA-like homologs (Fig. S2B). Our data from TIE-1 has established that the N-terminal region of PioA is important to produce a functional iron oxidoreductase (holo-PioA_C_) and, hence, the holo-PioA_C_B complex. To investigate the functional parallels of holo-PioA synthesis and PioAB complex formation in phototrophs, we studied Rhodomicrobium vannielii and Rhodomicrobium udaipurense. Both of these organisms have PioABC homologs. R. vannielii can perform phototrophic Fe(II) oxidation (52) while the ability of R. udaipurense to oxidize Fe(II) is unknown. The ability of R. vannielii and R. udaipurense to perform phototrophic EEU is also unknown.

10.1128/mBio.02668-19.6 FIG S6 Mass spectrometry (MS) analysis of the >170 kDa heme-stainable bands from R. vannielii DSM 162 and R. udaipurense JA643. The sequences of PioA and PioB homologs from R. vannielii (A) and R. udaipurense (B) are shown. Highlighted peptides (yellow) are the peptides detected by MS. The CXXCH motifs are shown in red. The first three residues of HR that contains a PioA proteolysis site between Val242 and Ala243 in TIE-1 are highlighted in green. Download FIG S6, PDF file, 0.9 MB. Copyright © 2019 Gupta et al. 2019 Gupta et al. This content is distributed under the terms of the Creative Commons Attribution 4.0 International license.

First, we studied the ability of R. vannielii DSM 162 (53) and R. udaipurense JA643 (54) to oxidize Fe(II) and perform phototrophic EEU. Both R. vannielii DSM 162 and R. udaipurense JA643 oxidized Fe(II) in cell suspension assays (Fig. 7B, chartreuse green and teal blue, respectively). We also observed that these phototrophs, similar to TIE-1, can perform phototrophic EEU from an electrode poised at +100 mV versus SHE in a μ-BEC reactor (Fig. 7C and Table S1) (14). Photoautotrophically grown R. vannielii DSM 162 and R. udaipurense JA643 with hydrogen were used for cell suspension assays, and their ability to oxidize Fe(II) suggests that the iron oxidase (PioA homologs) is produced under this growth condition. To investigate whether these phototrophs, like TIE-1, also synthesize holo-PioA_C_ and holo-PioA_C_B complex, cell fractions of photoautotrophically grown R. vannielii DSM 162 and R. udaipurense JA643 were analyzed. Heme staining of the soluble and membrane fractions from these bacteria showed band patterns similar to those of TIE-1. We observed two heme-stainable bands, an ∼34-kDa band in the soluble fraction and a >170-kDa band in the membrane fraction (Fig. 7D and E), for both phototrophs. The >170-kDa bands from R. vannielii DSM 162 and R. udaipurense JA643 were confirmed to contain PioA and PioB homologs by mass spectrometry analysis (Fig. S6). Interestingly, these bacteria also contain a smaller holo-PioA (∼34 kDa) in the soluble fraction than expected (∼48 kDa) from their annotated sequences (Fig. 7D). Furthermore, only the C-terminal peptides of the PioA homologs were detected by the mass spectrometry of these heme-stainable PioAB complexes (>170 kDa). Together, these results suggest that the PioA-like homologs in R. vannielii DSM 162 and R. udaipurense JA643, similar to the PioA of TIE-1, undergo postsecretory proteolysis of the N-terminal region to produce a smaller holo-PioA_C_.

### A holo-PioA_C_B complex as the cornerstone for photoferrotrophy and phototrophic EEU.

**FIG 8:**
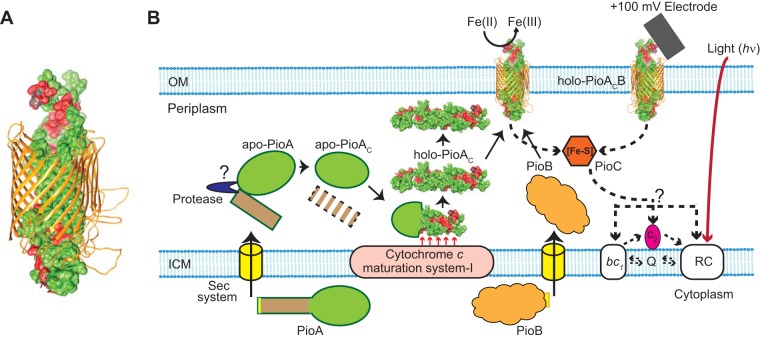
Proposed model for PioA_C_B complex synthesis and function. (A) *In silico*-predicted model for holo-PioA_C_B complex. (B) A model for assembly of the active holo-PioA_C_B complex. The model describes the postsecretory steps in the synthesis of holo-PioA_C_ and demonstrates an electron uptake mechanism via PioA_C_B complex that enables R. palustris TIE-1 to utilize both Fe(II) and poised electrodes that mimic insoluble iron minerals as electron donors for photosynthesis. PioC, a periplasmic high-potential iron-sulfur (Fe-S) protein, shuttles electrons from the inner face of the holo-PioA_C_B complex to photosynthetic reaction center. *In silico*-predicted models in panel A and in Fig. S7 in the supplemental material were used to demonstrate folded forms of holo-PioA_C_, PioB, and the holo-PioA_C_B complex in this model. Solid arrows, steps in protein transport, processing, and complex formation; dashed arrows, the path of electron transfer; OM, outer membrane; ICM, inner cytoplasmic membrane; Q, ubiquinone pool; *bc_1_*, *bc_1_* complex; RC, photosynthetic reaction center.

Our biochemical and genetic studies show that the holo-PioA_C_B complex is responsible for extracellular oxidation and electron uptake from both soluble Fe(II) and poised electrodes (that mimic insoluble iron minerals). The molecular mechanism of electron transfer by a porin and a single decaheme protein, as in the holo-PioA_C_B complex in TIE-1, is not well understood. To investigate whether the dimensions of predicted models of holo-PioA_C_ and PioB could support the formation of a holo-PioA_C_B complex to allow EEU to occur across the OM, we performed *in silico* analysis of PioA_C_ and PioB using RaptorX, a web-based server for protein structure prediction (55). The predicted structure of holo-PioA_C_ has the dimensions of ∼100 by 40 by 30 Å (Fig. S7A and B), and the predicted structure of PioB has an estimated pore diameter of ∼30 to 40 Å (Fig. S7C and D). Although cryo-electron microscopy (cryo-EM) or crystal structures will be necessary to validate the topology and structure of PioA_C_B, these *in silico* structure predictions suggest a holo-PioA_C_B model (Fig. 8A and Fig. S7E) where holo-PioA_C_ inserts through the entire length of PioB and supports EEU across the OM (∼40 to 50 Å in width) of TIE-1.

10.1128/mBio.02668-19.7 FIG S7 RaptorX-generated models of PioA_C_ and PioB. (A) Predicted model of PioA_C_ (green) and dodecaheme cytochrome *c* GSU1996 (PDB accession number 3OV0, best template used in the model preparation; brown) were overlaid in Chimera using default parameters. (B) Surface of the PioA_C_-model from 1st CXXCH to the 10th CXXCH. CXXCH motifs (1 to 10) are shown in red, and the rest of the protein is shown in green. (C and D) Predicted model of PioB showing dimensions of protein surface (C) and pore (D). The N-terminal region of PioB which forms a plug-like structure is not shown in this model for simplicity. These *in silico* models support the hypothesis that PioA_C_ can insert entirely through PioB and that the complex (E) can take up electrons from the extracellular electron donors across the OM. Download FIG S7, PDF file, 2.7 MB. Copyright © 2019 Gupta et al. 2019 Gupta et al. This content is distributed under the terms of the Creative Commons Attribution 4.0 International license.

### Conclusions.

The results support a model (Fig. 8B) for the synthesis and function of key proteins involved in photoferrotrophy and phototrophic EEU whereby the cytoplasmic PioA precursor (540 aa) is exported to the periplasm via the Sec pathway with the concomitant removal of its signal peptide (1 to 40 aa) (56). The periplasmic apo-PioA (500 aa) is then proteolytically processed at the Ala243 site by a periplasmic/membrane protease to facilitate heme attachment and produce holo-PioA_C_ (297 aa, 34 kDa). Holo-PioA_C_ exists in two forms, in a free periplasmic form and in a complex with PioB. The free periplasmic form of holo-PioA_C_ is required for the stability and incorporation of PioB in the OM. Based on the dependency of MtrB on MtrA for its OM localization (21, 47) and the discovery of MtrB with an opposite orientation in the OM (57), it was proposed that the folding and insertion of MtrB to the OM follow a different pathway than a typical β-barrel OM porin in *Shewanella* (57). Here, we propose that the orientation of PioB (Fig. 8 and Fig. S7) and its folding and insertion to the OM in TIE-1 are similar to those of MtrB. The holo-PioA_C_B complex catalyzes electron uptake from both extracellular electron donors such as Fe(II) and poised electrodes (that mimic insoluble iron minerals). Subsequently, electrons from the holo-PioA_C_B complex are transferred to the photosynthetic reaction center in the inner cytoplasmic membrane, most likely via PioC (periplasmic soluble iron-sulfur protein), as previously proposed (33, 34).

Although the 500-residue apo-PioA (54 kDa) is most likely secreted into the periplasm, we did not detect this apo-PioA in TIE-1. This lack of detection could be due to the rapid proteolysis of apo-PioA in TIE-1. In E. coli, we observed both the 54-kDa apo-PioA and the 34-kDa holo-PioA_C_^r^ (Fig. 1B and C), detecting heme attachment only in the 34-kDa C-terminal PioA. These results suggest that the proteolysis of apo-PioA occurs before heme maturation in both TIE-1 (and other photoferrotrophs) and E. coli. We could not detect a PioAB complex containing full-length apo-PioA; so heme maturation of PioA is likely important for formation of the PioAB complex. Our data demonstrate that the deletion of the N-terminal 200-aa region of apo-PioA does not abrogate production of holo-PioA_C_ in TIE-1. However, it causes a decrease in the cellular concentration of holo-PioA_C_ and holo-PioA_C_B complex, likely leading to a lag in photoferrotrophy (Fig. 2C) and an overall reduction in phototrophic EEU (Fig. 6A to C). The 200-aa region of the N terminus of PioA may play a role in maintaining the concentration of holo-PioA_C_ in TIE-1 at wild-type levels. This region could control the C-terminal PioA secretion rate and/or access to the cyt *c* maturation pathway (i.e., CcmF/H synthetase) (58, 59). The exact function of the N-terminal region will require further investigation. Although the Δ*pioB* mutant can produce soluble holo-PioA_C_, it did not oxidize Fe(II) (Fig. 5A and B) or perform phototrophic EEU (Fig. 6A to C). This indicates that both of these processes in TIE-1 are catalyzed extracellularly by holo-PioA_C_B complex (33, 60).

The PioAB system potentially catalyzes electron transfer from extracellular solid substrates, and its broad distribution suggests that this phenotype may also be widespread (15, 21). Interestingly, we found that PioA-like (decahemes with extended N termini) homologs are conserved only in phototrophic bacteria (Fig. 7A and Fig. S2). Although MtrA-like decaheme homologs and the MtrCAB electron conduit have been previously characterized, these proteins are found in nonphototrophic organisms such as *Shewanella* that use extracellular minerals as their terminal electron acceptors. Typically, these proteins allow electrons produced inside the cytoplasm to be transferred to the terminal electron acceptor. However, the MtrCAB conduit in *Shewanella* requires its extracellular decaheme component, MtrC, or its paralogues (OmcA and MtrF) to reduce Fe(III) or to transfer electrons to electrodes (23–25). In contrast, PioA-like homologs are found in phototrophic bacteria, and the PioAB conduit (with no apparent extracellular decaheme cyt *c* component) likely allow electrons to be transferred across the outer membrane from a variety of extracellular electron donors [such as soluble Fe(II), insoluble iron, and insoluble iron mineral proxies such as poised electrodes] (14). Subsequently, PioC transfers electrons from the PioAB conduit to the photosynthetic reaction center (33, 34). The transferred electrons are utilized to produce NAD(P)H that is required for carbon fixation via the Calvin-Bassham-Benson cycle in TIE-1 (14).

## MATERIALS AND METHODS

### Bacterial strains and plasmids.

A complete list of strains, plasmids, and primers used in this study are described in Table S2 in the supplemental material. Bacteria were grown using medium and culture conditions as previously described (14, 15), and details are provided in Text S1.

10.1128/mBio.02668-19.9 TABLE S2 Relevant strains, plasmids, and primers employed in this study. Download Table S2, DOCX file, 0.02 MB. Copyright © 2019 Gupta et al. 2019 Gupta et al. This content is distributed under the terms of the Creative Commons Attribution 4.0 International license.

10.1128/mBio.02668-19.10 TEXT S1 Supplemental methods. Download Text S1, DOCX file, 0.02 MB. Copyright © 2019 Gupta et al. 2019 Gupta et al. This content is distributed under the terms of the Creative Commons Attribution 4.0 International license.

### Fractionation and preparation of soluble and membrane fractions of TIE-1.

Photoautotrophically grown R. palustris TIE-1 strains with hydrogen in fresh water (FW) medium (10) were used for fractionation. Fractionation was done as previously described (6) with some modifications (see Text S1 in the supplemental material for a complete description of the methods used).

### Protein expression and purification.

Affinity purification of proteins from E. coli and R. palustris TIE-1 were performed as previously described (61). E. coli RK103 and the R. palustris TIE-1 Δ*pioA* mutant were used as the expression hosts (see Text S1 in the supplemental material for a complete description of the methods used).

### Antibody production and immunoblots.

Antibody production and immunoblotting are described in detail in Text S1.

### Cell suspension assay.

All cell suspensions were performed in an anaerobic chamber (80% N_2_, 15% CO_2_ and 5% H_2_; Coy Laboratory, Grass Lake, USA) at room temperature as previously described (6, 28, 62). TIE-1 and other strains were inoculated from a prephotoautotrophic culture in FW medium with hydrogen (80% H_2_, 20% CO_2_) and grown to an OD_660_ of ∼0.3. The cells were harvested by centrifugation (10,000 × *g* for 5 min), washed three times with HEPES buffer (50 mM HEPES, pH 7.0, 20 mM NaCl), resuspended, and concentrated to an OD_660_ of ∼0.9 in HEPES buffer supplemented with 1 mM Fe(II) and 5 mM nitrilotriacetic acid (NTA). One hundred microliters of the cell suspensions was aliquoted in the 96-well plate. To start the assay, the plate was placed under a 60-W incandescent light at a distance of 25 cm. Fe(II) measurement at the initial time point (time zero [*T*_0_]) was taken before the light source was turned on. All of the Fe(II) measurements were performed using a ferrozine assay (63).

### RNA-preparations and RT-qPCR.

RNA-preparations and reverse transcription-quantitative PCR (RT-qPCR) were done as previously described (14, 15), and details are provided in Text S1.

### Microfluidic bioelectrochemical cell and conditions.

The microfluidic bioelectrochemical cells (μ-BECs) were assembled and used as previously described (14) (see Text S1 in the supplemental material for a complete description of the methods used).
