# Supplementary material for: Photoferrotrophs Produce a PioAB Electron Conduit for Extracellular Electron Uptake
Source: mBio. 2019 Nov 5;10(6):e02668-19. doi: 10.1128/mBio.02668-19 (PMC6831781; doi:10.1128/mBio.02668-19)
Supplement: FIG S1 [file mBio.02668-19-sf001.pdf]

```
PioA            EPAMVGHTALPHDGQVGAVDPSVNALVDYVRGLQSAGKTEQNFTPVPAHGLALGDGHGGG      60
MtrA            ------------------------------------------------------------      0
MtoA            ------------------------------------------------------------      0

PioA            HGGISGHGASAHGSPHGAAAAGPRFDTRFAGHGSTENLLALPQIDGGPQLEQSLRFTASL      120
MtrA            ------------------------------------------------------------      0
MtoA            ------------------------------------------------------------      0

PioA            RLTTLSEKRILRSHQTRHTLAARLAREPDYVAEMPEPVRDAATVTTWVALENFDDTIEQP      180
MtrA            ------------------------------------------------------------      0
MtoA            ------------------------------------------------------------      0

PioA            KQFDFIEILAAKADDSRPVLMVATREP-RAAAAGR------APMVAPAPGDPDGRYFVGS      233
MtrA            -------------------LVVTPNAYASKWDEKMTPEQVEATLDKK-FA-EGNYSPKGA      39
MtoA            -------------ADEQPAAQSAPAAEAQPASQSQLPD---LSMEAKAPQ-TAQESLKRD      43
                                      :                    :

PioA            KPCETCHAGL-----FDEFQQTVMGRNIKSGKVTPQGKMECETCHGPGSAHVNGGGGRE-      287
MtrA            DSCLMCHKKSEK--VMDLFKG-VHG--AIDSSKSPMAGLQCEACHGPLGQHNKGG-----      89
MtoA            AVCTRCHDESETTPILAIYQT-KHG--F----RGDMRTPNCQTCHGESANHLKGNVDGKG      96
                  *  **        :  ::    *              :*::***  . * :*.

PioA            -KGGIRSFRPTDSRGFDVAEANSVCLSCHEKGDQTYWQGSQHETRGLACVNCHTVMRKVS      346
MtrA            -NEPMITFGKQSTLS--ADKQNSVCMSCHQDDKRMSWNGGHHDNADVACASCHQVHVAKD      146
MtoA            RPAPDVVFKKHTFPASDDKVRSAQCLTCHKGTNRTNWAGSAHQSNQMACNDCHKIHAKAD      156
                       *      .      .: *::**:  .:  * *. *:.  :** .** :    .

PioA            PRNQLKTVQVMDTCFQCHKDRKAQVQRSSHMPIRETKITCVNCHNPHGSATEKLLREATV      406
MtrA            PV--LSKNTEMEVCTSCHTKQKADMNKRSSHPLKWAQMTCSDCHNPHGSMTDSDLNKPSV      204
MtoA            TV--RERATQTEVCYTCHKERRADAHKISTHPIEAGKVVCSDCHNPHGSAGPKLLKKNTV      214
                     .     :.*  **..::*: :: *  *:.  ::.* :*******   . *.: :*

PioA            NDTCYTCHADKRGPFLFEHPPVRENCLNCHEPHGSNHESLLIVARQRLCQQCHTNPHNQP      466
MtrA            NDTCYSCHAEKRGPKLWEHAPVTENCVTCHNPHGSVNDGMLKTRAPQLCQQCHASDGHAS      264
MtoA            TETCFTCHADKRGPFLFAHQPVTEDCTNCHMPHGSNIAPLLKTRPPFMCQECHDGSHAS-      273
                .:**::***:**** *: * ** *:* .** ****    :* .    :**:** .

PioA            GLP-----------------------TSARWAVGNACQNCHNNIHGSNAPSGSRWHR 500
MtrA            NAYLGNTGLGSNV-------------GDNAFTGGRSCLNCHSQVHGSNHPSGKLLQR 308
MtoA            GTAVGPNAAGYQAGLSTINAAGTGA                                 330
                .                                *.:* *** ::**** *:*   :*
```
