# Supplementary material for: Photoferrotrophs Produce a PioAB Electron Conduit for Extracellular Electron Uptake
Source: mBio. 2019 Nov 5;10(6):e02668-19. doi: 10.1128/mBio.02668-19 (PMC6831781; doi:10.1128/mBio.02668-19)
Supplement: FIG S2 [file mBio.02668-19-sf002.pdf]

A

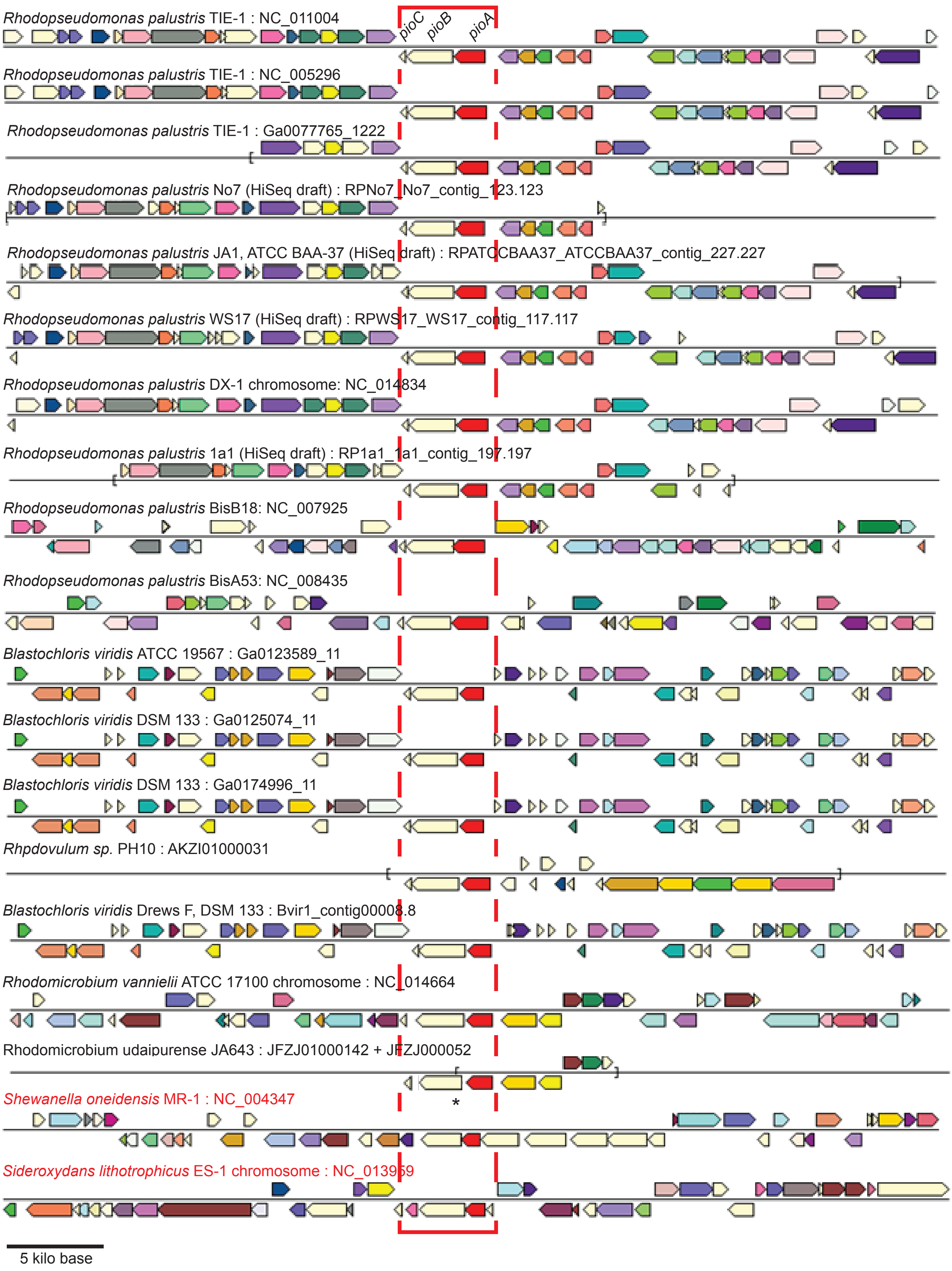

B

```
Rpal_TIE-1      ------MGGSRGAAAARDQSRRGRRSWISSLFVSIALAWGTWPAGAEPAMVGHTALPHDG 54
Rvan            MFFSILAGSSSGEPERGRLR------------LPLRAIWTLPTIGFLIAMTAGQ--ATAG 46
Ruda            MFFSILAGSSSGEPERRRLR------------LPLRAIWTLPAIGFLIAMASGQ--ATAG 46
Sone_MR-1       ------------------------------------------------------------ 0
Slit_ES-1       ------------------------------------------------------------ 0

Rpal_TIE-1      QVGAVDPSVNALVDYVRGLQSAGKTEQNFTPVPAH--GLALGDGHGGGHGGISG---HGA 109
Rvan            QTAAIGSD---V--FVYAMQAQ----AGEKPLPAGHPDVSSMESAGGAHGAKSAHSGKSA 97
Ruda            QTAAIGSD---V--FVYAMQAQ----AGEKPLPVGHPDVSSMGPAG-------AHSAKSA 90
Sone_MR-1       ------------------------------------------------------------ 0
Slit_ES-1       ------------------------------------------------------------ 0

Rpal_TIE-1      SAHGSPHGAAAAGPRFDTRFAGHGSTENLLALPQIDGGPQLEQSLRFTASLRLTTLSEKR 169
Rvan            HSAKSPHGGMGASPHAGLPSSKASPAKALLN------------HALFTPEAGL------- 138
Ruda            HGAKSPHGAMGASPHAGISSSKASPAKALLN------------HALFTPEAGL------- 131
Sone_MR-1       ------------------------------------------------------------ 0
Slit_ES-1       ------------------------------------------------------------ 0

Rpal_TIE-1      ILRSHQTRHTLAARLAREPDYVAEMPEPVRDAATVTTWVALENFDDTIEQPKQFDFIEIL 229
Rvan            ---------------------VGGSQRSSSN------------ADAET-----EHLIMVA 160
Ruda            ---------------------VGGAKRSSSN------------ADAET-----EHLIMVA 153
Sone_MR-1       -------------------------MKNCL---------KMKNL---------LPALTIT 17
Slit_ES-1       -------------------------MKPLR---------KIMVL---------SAFITGM 17
                                          .                             :

Rpal_TIE-1      AAKADDSRPVLMVATREPRAAAAGRAPMVAPAPG------------DPDGR-YFVGSKPC 276
Rvan            ESVPAAKAPAPGGKAKAADAAADSKAKVVRPAPD------------DPEGR-YYVGSEPC 207
Ruda            ESVPAAKAAP--------AAAADSQAKMIRPAAD------------DPEGR-YYVGSEPC 192
Sone_MR-1       M-----AMSAVMALVVTPNAYASKWDEKMTPEQV----EATLDKK-FAEGNYSPKGADSC 67
Slit_ES-1       A-----AMPCLMAADEQPAAQSAPAAEA-QPASQSQLPDLSMEAKAPQTAQESLKRDAVC 71
                                   * :        *                  ..        *

Rpal_TIE-1      ETCHAGLF-----DEFQQTVMGRNIKSGKVTPQGKMECETCHGPGSAHVNGGGGREKG-- 329
Rvan            KVCHAYLF-----DEFKFTVMGRNFEAGKDTPKGKMDCETCHGPASGHVNGGGGRLGG-- 260
Ruda            KVCHAYLF-----DEFKLTVMGRNFHAGKDTPKGKMDCETCHGPASAHVNGGGGRLGG-- 245
Sone_MR-1       LMCHKKSEK--VMDLFKG-V--HGAIDSSKSPMAGLQCEACHGPLGQHNKGG------NE 116
Slit_ES-1       TRCHDESETTPILAIYQT-K--HGF----RGDMRTPNCQTCHGESANHLKGNVDGKGRPA 124
                  **           ::     :.            :*::***  . * :*.

Rpal_TIE-1      GIRSFRPTDSRGFDVAEANSVCLSCHEKGDQTYWQGSQHETRGLACVNCHTVMRKVSPRN 389
Rvan            GIRSFRKSDPRT-SVADTNGVCLQCHEKNDRTYWKGSTHETRDVACTDCHTVMRKTSPRF 319
Ruda            GIRSFRKSDPRT-SVADTNGVCLQCHEKNDRTYWKGSTHETRDVACTDCHTVMRKTSPRF 304
Sone_MR-1       PMITFGKQSTLS--ADKQNSVCMSCHQDDKRMSWNGGHHDNADVACASCHQVHVAKDPV- 173
Slit_ES-1       PDVVFKKHTFPASDDKVRSAQCLTCHKGTNRTNWAGSAHQSNQMACNDCHKIHAKADTV- 183
                    *             .. *: **:  .:  * *. *:.  :** .** :    .

Rpal_TIE-1      QLKTVQVMDTCFQCHKDRKAQVQRSSHMPIRETKITCVNCHNPHGSATEK-LLREATVND 448
Rvan            QLAKGTVQDTCFQCHKDRRAQSLRTAHMPMIEGKISCSSCHNPHGSASETAMLKEATVND 379
Ruda            QLAKGTVQDTCFQCHKDRRAQSLRSAHMPMIEGKISCSSCHNPHGSASETAMLKEATVND 364
Sone_MR-1       -LSKNTEMEVCTSCHTKQKADMNKRSSHPLKWAQMTCSDCHNPHGSMTDS-DLNKPSVND 231
Slit_ES-1       -RERATQTEVCYTCHKERRADAHKISTHPIEAGKVVCSDCHNPHGSAGPK-LLKKNTVTE 241
                        :.*  **..::*:  : :  *:    :: * .*******   .  *.: :*.:

Rpal_TIE-1      TCYTCHADKRGPFLFEHPPVRENCLNCHEPHGSNHESLLIVARQRLCQQCHTNPHNQP-- 506
Rvan            TCYQCHADKRGPFLFEHPPVRENCMNCHEPHGSMHNSLLVVSRPRLCQRCHTAPHDVASL 439
Ruda            TCYQCHADKRGPFLFEHAPVRENCMNCHEPHGSMHNSLLVVARQRLCQRCHTGGFHPGTI 424
Sone_MR-1       TCYSCHAEKRGPKLWEHAPVTENCVTCHNPHGSVNDGMLKTRAPQLCQQCHASDGHASNA 291
Slit_ES-1       TCFTCHADKRGPFLFAHQPVTEDCTNCHMPHGSNIAPLLKTRPPFMCQECHDGSHAS-GT 300
                **: ***:**** *: * ** *:* .** ****    :* .    :**.**

Rpal_TIE-1      ---------------------GLPTSARWAVGNACQNCHNNIHGSNAPSGSRWHR      540
Rvan            G------------------QTTVTNNNRRIVSQACQNCHTNIHGSNAPSGSRWHR      476
Ruda            GLGV-----------TPADGGTLANNNRRLVGQACQNCHTNIHGSNAPSGSRWHR      468
Sone_MR-1       YLGNTGLGSNV-------------GDNAFTGGRSCLNCHSQVHGSNHPSGKLLQR      333
Slit_ES-1       AVGPNAAGYQAGLSTINAAGTGALYPSANNVGNACMNCHRQIHGSNSPAGGYLQR      355
                                               ..:* *** ::**** *:*   :*
```
