# Supplementary material for: Photoferrotrophs Produce a PioAB Electron Conduit for Extracellular Electron Uptake
Source: mBio. 2019 Nov 5;10(6):e02668-19. doi: 10.1128/mBio.02668-19 (PMC6831781; doi:10.1128/mBio.02668-19)

A

PioA

MGGSRGAAAARDQSRRGRRSWISSLFVSIALAWGTWPAGAEPAMVGHTALPHDGQVGAVDPSVNAL
VDYVRGLQSAGKTEQNFTPVPAHGLALGDGHGGGHGGISGHGASAHGSPHGAAAAGPRFDTRFAG
HGSTENLLALPQIDGGPQLEQSLRFTASLRLTTLSEKRILRSHQTRHTLAARLAREPDYVAEMPEPVRD
AATVTTWVALENFDDTIEQPKQFDFIEILAAKADDSRPVLMVATREPRAAAAGRAPMVAPAPGDPDGR
YFVGSKPCETCHAGLFDEFQQTVMGRNIKSGKVTPQGKMECETCHGPGSAHVNGGGGREKGGIRS
FRPTDSRGFDVAEANSVCLSCHEKGDQTYWQGSQHETRGLACVNCHTVMRKVSPRNQLKTVQVMD
TCFQCHKDRKAQVQRSSHMPIRETKITCVNCHNPHGSATEKLLREATVNDTCYTCHADKRGPFLFEH
PPVRENCLNCHEPHGSNHESLLIVARQRLCQQCHTNPHNQPGLPTSARWAVGNACQNCHNNIHGSN
APSGSRWHR

PioB

MAFRQFLLTSAAISVVTVIPLSATAETVAKEEPARSVPDFDAAHGGWSYSGEFEAGWRSFIQRPPKSAT
PWVSPTNPTGGGDRNNRSKFEEYGRIAPGFYAEYLRMTLQTKDGTYWNELRADNIGNNNQRYIFDFS
KAGEHYLTLGWDEIPHLYNTSALSIWNGVGTTTLTTPVAIPGNTLSIVAPATTTTAAQRAAVTNALAGKT
NLIDVGIDRRKGSAAYRWTPDPNWDVKASYSHEQREGTQIAGFPIGGLAGTGLQQMQAPRPIDDTTQI
GKLSGQYFGPTPWGGHYNVQVGGGFSLYDNSFNSFTVQNPFFDPANPRVFSPAARISLMPSNQAYN
TGVTTGIDLPFKTRWNSTFQYTTMRQNEAFMPFTVNPNITTFLLPASSLNGEVNTALYNTTATTQWTPE
FRTTTRYRYYDNDNQTPELLMPNYVVEDSSATGIAAGIPRRSLSMSYTKQNASGEMQWRPAKWVTF
GSTFGWEQWDRSRRDANVTNEFIGKLTADFKVHDIATLRTSGQYSERRYDNYDGLAMARDTYYSATA
NNGTNLLMRKFDMANRNQTKANAFLDISGPAGSVFRDFVISPTAGLRFEDYPDDPLFFGLKKSNTWNA
GIDITYSFTPGSSIQASYLYETYDRFQVGSGTLTNSATGLPNATPINAFGSNTLEKVQTILIASTLELIPGV
LDFKLGYAISFSHEDWDYGPYNGYALLANAGGAVYRPFPTVSTNYQRLDASLKHTIDPSIVAKLGWTG
EVYVKARYIWERNSVDNWQQDLMSPYLYLVDTTLARMIDMGATNPNYDAQYFQVSLNAKW

B

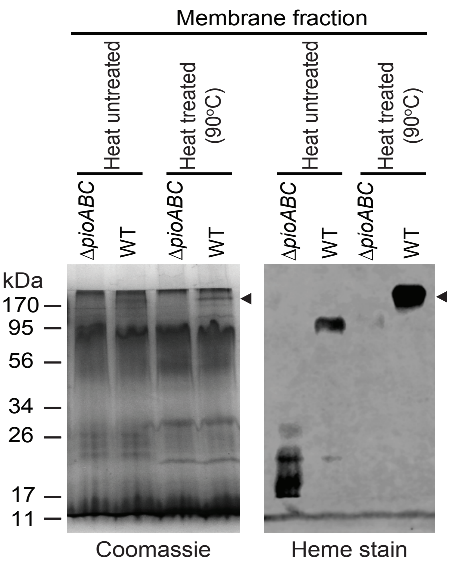

Supplement: FIG S4 [file mBio.02668-19-sf004.pdf]
