# Supplementary figures and images for: Photoferrotrophs Produce a PioAB Electron Conduit for Extracellular Electron Uptake
Source: mBio. 2019 Nov 5;10(6):e02668-19. doi: 10.1128/mBio.02668-19 (PMC6831781; doi:10.1128/mBio.02668-19)

**A**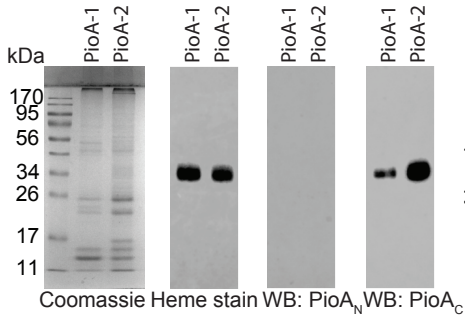**B**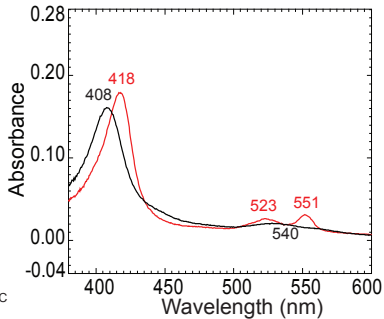

Supplement: FIG S3 [file mBio.02668-19-sf003.pdf]

**A**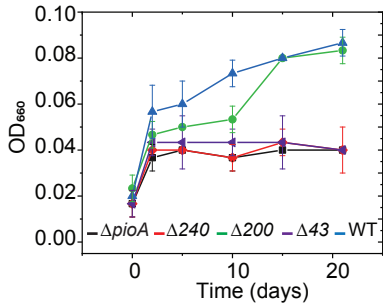**B**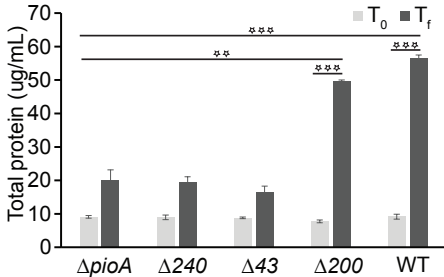

Supplement: FIG S5 [file mBio.02668-19-sf005.pdf]

**A**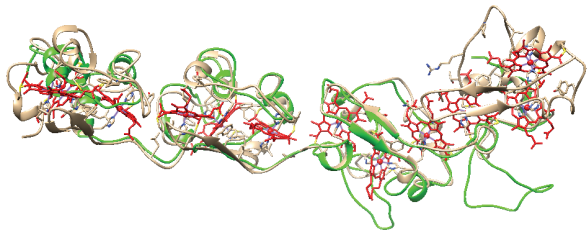**B**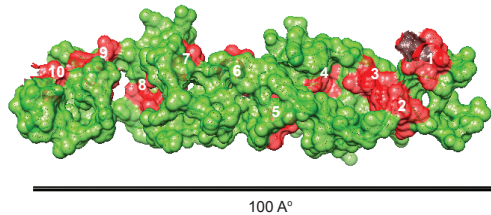**C**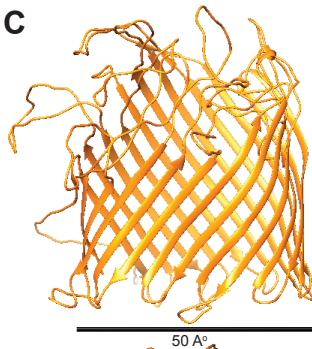**E**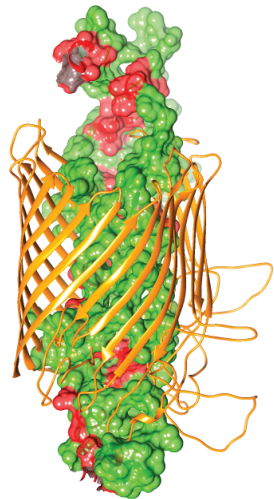**D**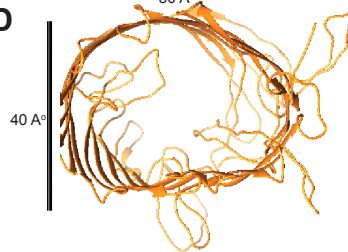

Supplement: FIG S7 [file mBio.02668-19-sf007.pdf]
