# Supplementary material for: Photoferrotrophs Produce a PioAB Electron Conduit for Extracellular Electron Uptake
Source: mBio. 2019 Nov 5;10(6):e02668-19. doi: 10.1128/mBio.02668-19 (PMC6831781; doi:10.1128/mBio.02668-19)
Supplement: TABLE S1 [file mBio.02668-19-st001.docx]

**Table S1:** Average maximum current uptake (nA cm^-2^) under continuous light in µ-BECs for different bacterial strains.

| Current uptake under continuous light for *R. palustris* TIE-1 and mutants represented in Fig. 6C | | | | | |
| --- | --- | --- | --- | --- | --- |
|  | Replication-1 | Replication-2 | Replication-3 | Average | s.d |
| FW media control | 0.1402 | 0.0902 | 0.2431 | 0.1578 | 0.1134 |
| ∆*pioABC* | -1.2706 | -1.5231 | -1.1532 | -1.3156 | -0.3067 |
| ∆*pioA* | -9.8881 | -8.3282 | -12.4324 | -10.2162 | -2.9548 |
| ∆*pioB* | -0.3269 | -0.5442 | -0.4773 | -0.4495 | -0.2266 |
| ∆*200* | -231.248 | -268.3021 | -209.7632 | -236.4378 | -45.9456 |
| WT TIE-1 | -422.326 | -401.4381 | -440.7623 | -421.5088 | -27.3057 |
| Current uptake under continuous light (nAcm^-2^) for *R. palustris* TIE-1, *R. vannielii* DSM 162 and *R. udaipurense* JA643 represented in Fig. 7C | | | | | |
|  | Replication-1 | Replication-2 | Replication-3 | Average | s.d |
| FW media control | 0.0227 | 0.0284 | 0.0192 | 0.0234 | 0.0046 |
| WT TIE-1 | -398.727 | -387.408 | -369.72 | -385.285 | 14.6196 |
| *R. vannielii* DSM 162 | -82.513 | -129.202 | -93.831 | -101.848 | 24.3552 |
| *R. udaipurense* JA643 | -841.827 | -787.02 | -751.018 | -793.288 | 45.7279 |
