# Supplementary material for: Photoferrotrophs Produce a PioAB Electron Conduit for Extracellular Electron Uptake
Source: mBio. 2019 Nov 5;10(6):e02668-19. doi: 10.1128/mBio.02668-19 (PMC6831781; doi:10.1128/mBio.02668-19)
Supplement: FIG S6 [file mBio.02668-19-sf006.pdf]

A

>PioA_*R. vannielii*

MFFSILAGSSSGEPERRRLRLPLRAIWTLPAIGFLIAMASGQATAGQTAAIGSDVFVYAMQAQAGEKPLPVGHPDVSSIGSVGGAHGA
KSAHGAKSPHGAMGASPHAGISSSKASPAKALLNHALFTPEAGLVGGAKRSSSNADAETEHLIMVAESVPAAKAAPAAAADSQAKMI
RPAADDPEGRYYVGSEPCKVCHAYLFDEFKLTVMGRNFHAGKDTPKGKMDCETCHGPASAHVNGGGGRLGGGIRSFRKSDPRTS
VADTNGVCLQCHEKNDRTYWKGSTHETRDVACTDCHTVMRKTSPRFQLAKGTVQDTCFQCHKDRRAQSLRSAHMPIEGKISCSS
CHNPHGSASETAMLKEATVNDTCYQCHADKRGPFLFEHAPVRENCMNCHEPHGSMHNSLLVVARQRLCQRCHTGGFHPGTIGLGV
TPADGGTLANNNRRLVGQACQNCHTNIHGSNAPSGSRWHR

>PioB_*R. vannielii*

MEFHGRIVLTSLLSIAVASIALQSARAGDKPAPAPVAPAADEKIELDTWGDIEFGGRYFPKKPSELGSNKWSTTTIQRPEAESIAKYEEY
GNVPQGFYFEKLTVGGQTKSGEYAAELRATDIGNNNQRYIFDWFVTGELSGTVTWDQTPHLFSTTALNIWNGVGTDHLTPKAQVGA
YTTTTPAYLASIQSLIAQGNTIRIGIERDRFEADQRWTPTPHWEIRGNYFYDHRYGTQIAGTNWGGGAGPVMVQMPRPVDDTTQNAK
VSGERSGEWAYGKYNVKITGGLSTFDNSFSSYTVENPFVATGTTCASAYTGTANAPCSLISLMPSNEAYSGNVTSAIDLPFKSRFMNT
VQYTSMRQNDPFQDPTGSTGNTYYIPGSTTIQVANAATSLNGEVNTLLVNNVLTTRITDDLKSTFRYRYYDNDNQTPVLTWNWVTED
GATSTATRRNFGYSYTKQNASGDLTYHVLKNASVGGSAGWERIDREKREALQTNEYIGKVYGDARWDDIGQLRASYQYSERRYDR
YDPKAWYDYLYPAYPQVVGGITYTQGGGGGGTNWGVRKLDLADRDREKAMVLFTFDNIPHVPNLSLTPSFGLRNDNYLTDPNRLVTI
AGTGSAAGNTYSTYEMGLLKDNSWNGGLEASYSFGPGLTVSAAYVREEFDKDLAGSTSTTTSGTGATGTANALDSINRWSSNMKE
NVNTFILGSNFIFNDRFDISASYSIALGNENWSTEALGATSVCNPVGGAGSNITGACQPIDPVKTTLQRIDLQSRFKLDSELVSQLGFEG
DVYWKLKYSLDRSRVKNWQNDLVTPYMYLVDNTARDIAMAAYNPNYDVHVVATSLNFKW

B

>PioA_*R.udaipurense*

MFFSILAGSSSGEPERRRLRLPLRAIWTLPAIGFLIAMASGQATAGQTAAIGSDVFVYAMQAQAGEKPLPVGHPDVSSMGPAGAHSA
KSAHGAKSPHGAMGASPHAGISSSKASPAKALLNHALFTPEAGLVGGAKRSSSNADAETEHLIMVAESVPAAKAAPAAAADSQAKMI
RPAADDPEGRYYVGSEPCKVCHAYLFDEFKLTVMGRNFHAGKDTPKGKMDCETCHGPASAHVNGGGGRLGGGIRSFRKSDPRTS
VADTNGVCLQCHEKNDRTYWKGSTHETRDVACTDCHTVMRKTSPRFQLAKGTVQDTCFQCHKDRRAQSLRSAHMPIEGKISCSS
CHNPHGSASETAMLKEATVNDTCYQCHADKRGPFLFEHAPVRENCMNCHEPHGSMHNSLLVVARQRLCQRCHTGGFHPGTIGLGV
TPADGGTLANNNRRLVGQACQNCHTNIHGSNAPSGSRWHR

>PioB-partial_*R.udaipurense*

PIADTTQNAKASGERSGDWAYGRYNVKITGGISTFENDLKSFTVENPFVAAGTTCASTFTAAANTPCSRISLMPSNEAYTGNMTAGID
LPFRSRFMNTVQYTAMRQNDSFQDPTVATGNTNTAVTGITNAATSLNGEVNALLVNNVLNTRITDDLKSTLRYRYYDNDNQTPVRTW
NWVSEDGAVGAVRQNFGYSYTKQNASGDLTYHVLKNASVGGSAGWERIDRDKREALRTDELIGKVYGDARWNDIGQLRASYQYSE
RRFDNYDPESWYKTLFPGGTGGNMNPWGMRKFDLADRDREKAMVLFTFDNIPHVPNLSLTPSFGLRNDNYLTDPNKILTDSAGNTT
TEMGLLKDNSWNAGIEGSYSFGPGLTVSAAYVREEFDKDLVGSLTTQDSATGIIGATTANRYFSNMKEDVDTFIVGTNFVFNDRFDIS
ASYSIALGKEDWTNKDIAGTTCAPTSTGANCAPWPTVNTTLQRIDAQARYKLDPELITQLGFEGDVFWKLKYSWDRSRVDNWQNDY
VTPYMYLVDNTARNISMASYNPNYDIHAVSTSLNFKW
