# Supplementary material for: Photoferrotrophs Produce a PioAB Electron Conduit for Extracellular Electron Uptake
Source: mBio. 2019 Nov 5;10(6):e02668-19. doi: 10.1128/mBio.02668-19 (PMC6831781; doi:10.1128/mBio.02668-19)
Supplement: TABLE S2 [file mBio.02668-19-st002.docx]

**Table S2**. Relevant strains, plasmids, and primers employed in this study.

| **Strain, plasmid** | **Description** | **Reference** |
| --- | --- | --- |
| *E. coli* | |  |
| WM6026 | [*lacI^q^ rrnB3* Δ*lacZ*4787 *hsdR514*Δ*araBAD567* Δ*rhaBA D568 rph-1 att*l∷pAE12(Δ*ori*R6K-*cat*∷Frt5) Δ*endA*∷Frt *uidA*(ΔMluI)∷*pir att*HK∷pJK1006Δ(*ori*R6K*cat*∷ Frt5;*trfA*∷Frt)]. Donor strain for conjugation. | W. Metcalf, UI,  Urbana-Champaign |
| DH10B | F^-^ *endA1* *recA1* *galE15 galK16 nupG rpsL* Δ*lacX74* Φ80*lacZ*Δ*M15* *araD139* Δ(*ara,leu*)*7697 mcrA* Δ(*mrr-hsdRMS-mcrBC*) λ ^‑^ . Used as standard cloning strain. | *Casadaban and Cohen, 1980* |
| RK103 | *E. coli* ∆*ccm*, Used as an expression strain for the *pioA* and *pioB genes* | *Feissner et al, 2006* |
| *R. palustris* TIE-1 | |  |
| Wild type (WT) | Isolated from Woods Hole, MA*.* | *Jiao et al, 2005* |
| *∆pioA* | *R. palustris* TIE-1 *∆pioA* | *Jiao and Newman, 2007* |
| *∆pioABC* | *R. palustris* TIE-1 *∆pioABC* | *Jiao and Newman, 2007* |
| *∆240* | *R. palustris* TIE-1 *∆240N-pioA* (gene encoding for 1-240 aa of PioA has been deleted from the chromosome) | This study |
| *∆200* | *R. palustris* TIE-1 *∆200N-pioA* (gene encoding for 44-240 aa of PioA has been deleted from the chromosome) | This study |
| *∆43* | *R. palustris* TIE-1 *∆43N-pioA* (gene encoding for 1-43 aa of PioA has been deleted from the chromosome) | This study |
| Complemented *∆pioA -1* | *∆pioA* TIE-1 expressing pDG09 (full-length *pioA* gene from WT *R. palustris* TIE-1 with a C-terminal HIS tag under constitutive promoter P*aphII*). Used for PioA analysis | This study |
| Complemented *∆pioA -2* | *∆pioA* TIE-1 expressing pDG10 (*pioA* gene lacking encoding sequence for 1-240 aa region from WT *R. palustris* TIE-1 with a C-terminal HIS tag under constitutive promoter P*aphII*). Used for PioA analysis | This study |
| Complemented *∆pioA -3* (cWT) | *∆pioA* TIE-1 expressing pDG11 (full-length *pioA* gene from WT *R. palustris* TIE-1 with a C-terminal HIS tag under the native *pio* promoter). Used for complementation assay | This study |
| Complemented *∆pioA -4* (c*240*) | *∆pioA* TIE-1 expressing pDG12 (*pioA gene* lacking encoding sequence for 1-240 aa region from WT *R. palustris* TIE-1 with a C-terminal HIS tag under the native *pio* promoter). Used for complementation assay | This study |
| Complemented *∆pioA -5* (c*200*) | *∆pioA* TIE-1 expressing pDG13 (pioA gene lacking encoding sequence for 43-240 aa region from *R. palustris* TIE-1 *∆200 mutant* with a C-terminal HIS tag under the native *pio* promoter). Used for complementation assay | This study |
| Others | |  |
| *R. vannielii* DSM 162 | Wild type | *Duchow and Douglas, 1949* |
| *R. udaipurense* JA643 | Wild type | *Tushar et al, 2014* |
| **Plasmid** | | |
| pRGK330 | pBad24, Cm^r^, Used for cloning and gene expression in *E. coli* | *Feissner et al, 2006* |
| pRGK332 | pBAD24-derived plasmid with C4-periplasmic signal peptide from *Bordetella pertussis* inserted after RBS site under p*BAD* promoter, Cm^r^. Used for cloning and gene expression in *E. coli* | *Feissner et al, 2006* |
| pGEX-4T-1 | This expression vector with an N-terminal GST fusion to the insert under the control of the IPTG-inducible Tac promoter, Amp^r.^ Used for cloning and gene expression in *E. coli* | Amersham Biosciences |
| pRGK333 | a pGEX-derived plasmid containing *CcmA-H* genes under pTac promoter, Amp^r^ | *Feissner et al, 2006* |
| pJQ200KS | Mobilizable suicide vector; *sacB*, Gm^r^ | *Quandt and Hynes, 1993* |
| pRhokS-2 | pRho broad-host-range expression vector with *aphII*, a constitutive promoter, Cm^r^, Km^r^. Used for cloning and gene expression under the constitutive promoter in *R. palustris* TIE-1 | *Katzke et al, 2010* |
| pAB307 | pBBR1MCS-5 (Kovach et al., 1995) derived plasmid containing the *pio* promoter of *R. palustris* TIE-1, Gm^r^. Used for cloning and gene expression under the native *pio* promoter in TIE-1 | *Bose and Newman, 2011* |
| pDG01 | The *B. pertussis* cyt *c_4_* periplasmic signal peptide was amplified from pRGK322 using C4S_F and C4S_R primers. Similarly, the *pioA* gene (Rpal_0817) lacking its predicted Sec signal peptide (1-40 aa) was amplified from *R. palustris TIE-1* with a C-terminal His tag using 41-540pioA_F and pioA_R primers. Then these two amplicons were fused by overlapping PCR using C4S_F and pioA_R. The fusion product ‘C4S-41-540pioA-His’ was cloned into pRGK332 using NheI and XbaI sites. Used for protein production in *E. coli* | This study |
| pDG02 | The *pioB* gene was amplified from *R. palustris* TIE-1 with its RBS site and C-terminal Flag tag using PioB_F and PioB_R primers. The amplicon was then cloned into pDG1 with BglII and XbaI sites. Used for protein production in *E. coli* | This study |
| pDG03 | The *pioA* gene encoding for the C-terminus PioA (241-540 aa) with a C-terminal His tag from *R. palustris* TIE-1 cloned into the NcoI and XbaI sites of pRGK330 using HRpioA_F/pioA_R primers. Used for protein production in *E. coli* | This study |
| pDG04 | C4 periplasmic signal peptide was amplified using C4S_F and C4S_R2 primers. Similarly, the *pioA* gene encoding for C-terminus PioA (241-540 aa) with a C-terminal His tag was amplified from *R. palustris TIE-1* using HRpioA_F2/pioA_R primers. Then these two amplicons were fused by overlapping PCR using C4S_F and pioA_R. The fusion product ‘C4-HRpioA-His’ cloned into the NheI and XbaI site of pRGK332. Used for PioA production *E. coli* | This study |
| pDG05 | The *pioA* gene encoding for the N-terminus region (41-240 aa) from *R. palustris* TIE-1 cloned into the BamHI and NotI sites of pGEX-4T-1 using GEX-Nter_F/GEX-Nter_R primers. Used for protein production in *E. coli* | This study |
| pDG06 | 1-kilo base (kb) upstream and 1 kb downstream region of the ∆240*pio*A gene from WT *R. palustris* TIE-1 cloned into the SacI and BamHI sites of pJQ200KS using Up_pioA_F/Up_pioA_R (upstream) and Dw_∆240_F/Dw_∆240_R (downstream) primers. Used for generating *R. palustris TIE-1* ∆240 mutant | This study |
| pDG07 | 1 kb upstream and 1 kb downstream region of the ∆43*pio*A gene from WT *R. palustris* TIE-1 cloned into the SacI and BamHI sites of pJQ200KS using Up_pioA_F/Up_pioA_R (upstream) and Dw_∆43_F/Dw_∆43_R (downstream) primers. Used for generating *R. palustris TIE-1* ∆43 mutant | This study |
| pDG08 | 1 kb upstream and 1 kb downstream region of the ∆200*pio*A gene from WT *R. palustris* TIE-1 cloned into the SacI and BamHI sites of pJQ200KS using Up_∆200_F/Up_∆200_R (upstream) and Dw_∆240_F/Dw_∆240_R (downstream) primers. Used for generating *R. palustris TIE-1* ∆200 mutant | This study |
| pDG09 | A full-length *pioA* gene from TIE-1 with a C-terminal His tag cloned into the NdeI and BamHI sites of pRhokS-2 using FlpioA_F/pioA_R primers. Used for protein production in *R. palustris* TIE-1 | This study |
| pDG10 | The *pioA* gene encoding for C-terminus PioA (241-540 aa) with a C-terminal His tag from WT *R. palustis* TIE-1 cloned into the NdeI and BamHI sites of pRhokS-2 using HRpioA_F2/pioA_R primers. Used for protein production *R. palustris* TIE-1 | This study |
| pDG11 | A full-length *pioA* gene with a C-terminal His tag from WT *R. palustis* TIE-1 cloned into the NdeI and BamHI sites of pAB307 replacing *lacZ*. FlpioA_F/pioA_R primers were used. Used in the complementation assay | This study |
| pDG12 | The *pioA* gene encoding for C-terminus PioA (241-540 a with a C-terminal His tag) from WT *R. palustis* TIE-1 cloned into the NdeI and BamHI sites of pAB307 using HRpioA_F2/pioA_R primers. Used in the complementation assay | This study |
| pDG13 | The *pioA* gene lacking 200 aa-region with a C-terminal His tag form ∆*200* mutant cloned into the NdeI and BamHI sites of pAB307 replacing *lacZ*. HRpioA_F/pioA_R primers were used. Used in the complementation assay | This study |

| **Oligonucleotide** | **Sequence (5' --> 3')** |
| --- | --- |
| Expression plasmids construction | |
| C4S_F (NheI) | AACCATTGGGCTAGCAGGAGGATTTCATGAAGCGTGTGC |
| C4S_R | GTCCGACCATCGCCGGTTCGGCGGCGAAACTCATGGAG |
| 41-540pioA_F | CTCCATGAGTTTCGCCGCCGAACCGGCGATGGTCGGAC |
| pioA_R (His tag_BglII, BamHI, XbaI) | AACATCTAGAGGATCCAGATCTCAATGGTGATGGTGATGGTGTCGGTGCCAGCGCGATCC |
| pioB_F (BglII) | GTGGAGATCTAGGAGGACTTCACCATGGCGTTTAGGCAATTCCTG |
| pioB_R (Flag tag, XbaI) | ATATTCTAGATTACTTGTCGTCATCGTCTTTGTAGTCCCATTTGGCGTTGAGCGAG |
| HRpioA_F (NcoI) | ATATCCATGGTCGCGACGCGCGAGC |
| C4S_R2 | TCGCGCGTCGCGACCATGGCGGCGAAACTCATGGAGTGAAC |
| HRpioA_F2 | TCACTCCATGAGTTTCGCCGCCATGGTCGCGACGCGCGAG |
| FlpioA_F (NdeI) | GGCAGAACATATGGGGGGCTCTCGGG |
| HRpioA_F2 (NdeI) | TCTCCATATGGTCGCGACGCGCGAG |
| GEX-Nter_F (BamHI) | TGGTGGATCCGAACCGGCGATGGTCGGAC |
| GEX-Nter_R (NotI) | ATATGCGGCCGCCAGGACCGGCCGGGAATC |
| Deletion plasmids construction | |
| UP_pioA_F (SacI) | ACCAGAGCTCTTCCAAACTGTTCGTTCG |
| UP_pioA_R (XbaI) | AGCATCTAGAGTCCGTCCCCCGCAAGTG |
| Dw_∆240_F (XbaI) | ATCATCTAGAATGGTCGCGACGCGCGAG |
| Dw_∆240_R (BamHI) | TGTAGGATCCTATTACGGTCACCACGGAG |
| Dw_∆43_F (XbaI) | ATCATCTAGAATGGTCGGACACACCGCCTTG |
| Dw_∆43_R (BamHI) | TGTAGGATCCAATAGGTCTGGTCGCCCTTCTCGTG |
| Up_∆200_F (SacI) | AGAGGAGCTCACATCGTGCTCAACGACCTCG |
| UP_∆200_R (XbaI) | ATCATCTAGATTCGGCGCCGGCTGGC |
| Plasmid sanger sequencing/ mutant confirmation | |
| cNKO_F | TCGACGTCGCCCACGACACGTG |
| cNKO_R | ACGAAGTAACGGCCATCGGGATCGC |
| pioA4B_L_F | ACCACGAGTCGCTGCTGATC |
| pioB_M_F | ACACTACAACGTCCAGGTCG |
| pioB_M_R | ATCAGGATGGTCTGCACTTTCTC |
| Seq_A-mid_F | TGTTCGACGAGTTCCAGCAGAC |
| Seq_A-mid_R | TCTGCTGGAACTCGTCGAACAG |
| Seq_pBAD_F | ACTCTCTACTGTTTCTCCATACCC |
| Seq_pBAD_F | CTTTACTAAGCTGATCCGGTGGATG |
| Pio_conf_F1 | ATTGCGAGTCGTCCTGTTGCAC |
| Pio_conf_R1 | TCTGCGGTGCTCTGGCGTG |
| Pio_conf_F2 | ACGGCGATCGATAGTGGTC |
| Pio_conf_R2 | TCACTATAGGGCGAATTGGAG |
| MidpioA_R | TCAGGCAGACGCTGTTGG |
| pJQ_seq_F | GTAGCGAGTCAGTGAGCGAGGAAGC |
| pJQ_seq_R | GACTCACTATAGGGCGAATTG |
| RT-qPCR | |
| pioA (Rpal_0817)-F | GTTACTTCGTCGGCTCCAAG |
| pioA_(Rpal_0817)-R | ACCTTGCCGGACTTGATG |
| *clpX* (Rpal_3308)-F | GGAGATCTGCAAGGTTCTCG |
| *clpX* (Rpal_3308)-R | CCGCTTGTAGTGATTGTGGA |
| *recA* (Rpal_4376)-F | ATCGGCCAGATCAAGGAAC |
| *recA* (Rpal_4376)-F | GAATTCGACCTGCTTGAACG |
