# Supplementary material for: Photoferrotrophs Produce a PioAB Electron Conduit for Extracellular Electron Uptake
Source: mBio. 2019 Nov 5;10(6):e02668-19. doi: 10.1128/mBio.02668-19 (PMC6831781; doi:10.1128/mBio.02668-19)
Supplement: TEXT S1 [file mBio.02668-19-s0001.docx]

**Supplementary Material**

**Title:** Photoferrotrophs produce a PioAB electron conduit for extracellular electron uptake

**Authors:** Dinesh Gupta^a^, Molly C. Sutherland^a^, Karthikeyan Rengasamy^a^, J. Mark Meacham^b,c^, Robert G. Kranz^a^, Arpita Bose^a^#

^a^Department of Biology, Washington University in St. Louis, St. Louis, MO, USA.

^b^Department of Mechanical Engineering and Materials Science, Washington University in St. Louis, St. Louis, MO, USA.

^c^Institute of Materials Science and Engineering, Washington University in St. Louis, St. Louis, MO, USA.

**#Address correspondence to** Arpita Bose

Campus Box 1137

One Brookings Drive, St. Louis, MO, 63130

Email: [abose@wustl.edu](mailto:abose@wustl.edu)

Phone: +1-314-935-6236

**Supplementary Materials and Methods**

**Media and culture conditions.** *Escherichia coli* strains were grown in Lysogeny broth (LB) or LB-agar at 37^o^C. Media was supplemented with appropriate combinations of antibiotics (50 µg/mL kanamycin, 20 µg/mL gentamycin, 100 µg/mL ampicillin, or 25 µg/mL chloramphenicol) as indicated for different constructs. Liquid cultures were grown with shaking at 200 rpm. *E. coli* WM3064 was supplemented with 2.4 mM diaminopimelic acid. *Rhodopseudomonas palustris* TIE-1 (TIE-1) strains were grown in YP broth (0.3% yeast extract; 0.3% peptone) supplemented with 10 mM succinate and 10 mM MOPS pH 7.0 (YPSMOPS) or YPSMOPS-agar at 30^o^C for aerobic chemoheterotrophic growth. Media was supplemented with antibiotics (200 µg/mL kanamycin or 400 µg/mL gentamycin) when necessary. The aerobic chemoheterotrophic cultures were grown in dark with shaking at 200 rpm. For anaerobic photoautotrophic growth, TIE-1 strains were grown on hydrogen (80% H_2_: 20% CO_2_ at 50 kPa) or 5 mM Fe(II) chloride in freshwater (FW) medium (1) supplemented with 20 mM sodium bicarbonate in sealed sterile serum bottles. The photoautotrophic culture with Fe(II) contained 10 mM nitrilotriacetic acid (NTA) to prevent iron precipitation (2) and the headspace atmosphere consists of 80% N_2_ and 20% CO_2_. Media was supplemented with antibiotics (200 µg/mL kanamycin or 400 µg/mL gentamycin) when necessary. The phototrophic cultures were grown without shaking at 20-30 cm distance from a 60 W incandescent light bulb. In all cases where a change in culture medium was required, cells were washed three times in basal FW medium post-centrifugation at 5,000 x *g*. *R. vannielii* DSM 162 and *R. udaipurense* JA643 were photoautotrophically grown with hydrogen in FW medium as described above for wild type TIE-1.

**Protein expression and purification from *Escherichia coli*.** *E. coli* RK103 was used as the expression host. To facilitate expression of heme-attached PioA (holo-PioA), we used an engineered *E. coli* RK103 (∆*ccm*) strain (3) where the *ccmA-H* genes are overexpressed through a plasmid (pSysI) under an IPTG-inducible promoter (3). To ensure correct localization of the protein in the *E. coli*’s periplasm, we replaced the PioA signal peptide with a validated signal peptide from the cytochrome *c*_4_ gene (3) and engineered a C-terminal 6XHis tag. Culture, induction, and affinity purification of protein was done as previously described (4). *E. coli* strains were cultured in LB (5 mL) supplemented with the appropriate antibiotics from a single colony and grown overnight at 37^o^C and 200 rpm. Starter cultures (100 mL) were inoculated with the 5 mL pre-grown cultures and cultivated in LB with antibiotics for ~18 h. The starter cultures were diluted 1/10 into 1 L of LB with antibiotics and grown to an OD_600_ of ~1.2 and then induced with 1 mM IPTG for pGEX-derived plasmids or 40 mM arabinose for pBAD-derived plasmids. After ~16 h of induction, cultures were harvested by centrifugation (10,000 x *g* for 30 min at 4^o^C) and stored at -80^o^C. Cell pellets were thawed on ice for ~30 min, resuspended in the appropriate buffer (GST buffer: 4.3 mM Na_2_HPO_4_ pH 7.3, 1.5 mM KH_2_PO_4_, 2.7 mM KCl, and 140 mM NaCl and His buffer: 20 mM Tris-HCl pH 7.2 and 100 mM NaCl) supplemented with 1 mM phenylmethanesulfonyl fluoride (PMSF; Sigma-Aldrich) and 1 mg/mL egg white lysozyme (Sigma-Aldrich), and shaken on ice for ∼30 min. Cells were lysed by sonication (6 × 30 sec on, 30 sec off) on ice using a Branson250 sonicator (50% duty, 6 output). The sonicate was cleared of cell debris by centrifugation (24,000 x *g* for 30 min) at 4^o^C. The soluble and membrane fractions were then separated by high-speed ultracentrifugation (100,000 x *g* for 45 min) at 4^o^C. Membrane pellets were solubilized in the appropriate buffer with 1% Triton X-100 (TX-100; Sigma-Aldrich) and affinity purified by a batch method with the appropriate resin [GST affinity tag−Glutathione agarose (Pierce) and His affinity tag−TALON Metal affinity resin (Clontech)]. Columns were washed by gravity flow and eluted in 4 mL of the appropriate buffer (20 mM L-glutathione in GST buffer and 150 mM imidazole in His buffer) with 0.02% TX-100. Purified protein was concentrated using 30 kDa Amicon protein concentrators, and total protein concentrations were determined by a Bradford assay (Sigma). Purifications used for redox titrations were subsequently buffer exchanged and concentrated in a 10 kDa Amicon filter to decrease imidazole concentration below 1 mM.

**Fractionation and preparation of soluble and membrane fractions of TIE-1.** Photoautotrophically grown *R. palustris* TIE-1 strains were used for fractionation. TIE-1 strains were grown with hydrogen in fresh water (FW) medium (1) (500-1,000 mL) to an OD_660_ ~1.2 before harvested by centrifugation (10,000 x *g* for 30 min) at 4^o^C and stored at -80^o^C. To mimic the photoferrotrophic condition, TIE-1 strains were first grown with hydrogen in FW medium (500 mL) to an OD_660_ ~1 and then exposed to ~3 mM Fe(II) chloride and 6 mM NTA (Nitrilotriacetic acid) under a headspace atmosphere of 80% N_2_ and 20% CO_2_. Cells were harvested when all Fe(II) was oxidized (T_f_ = T_~22h_) and stored at -80^o^C. Cell pellets were thawed on ice for ~30 min, resuspended in Tris-buffer (20 mM Tris-HCl pH 7.2, 100 mM NaCl) supplemented with 1 mM PMSF and 1 mg/mL egg white lysozyme, and then shaken on ice for ∼45 min. Cells were lysed by three passes at 18,000 lbf/in^2^ through a high-pressure homogenizer (Avestin EmulsiFlex C5, Canada). The lysates were centrifuged (24,000 x *g* for 30 min) at 4^o^C to separate out supernatant and pellet. The pellets were solubilized in Tris-buffer with 5% Triton X-100 (Sigma-Aldrich) and used as the membrane fraction. Supernatants were further centrifuged (100,000 x *g* for 45 min) at 4^o^C to separate out any remaining membrane fraction and resulting ultrasupernatant was used as a soluble fraction.

**Protein expression and purification from *R. palustris* TIE-1.** The *pioA* gene was expressed in the *R. palustris* TIE-1 ∆*pioA* background using a broad-host-range expression vector either under a constitutive promoter or the native *pio* promoter (Table S2). The *pioA*-expression plasmids were conjugated into the ∆*pioA* mutant using the mating strain *E. coli* WM3064 and selected on YPSMOPS agar with 400 µg/mL kanamycin (5). A single colony was grown in YPSMOPS broth (5 mL) with the appropriate antibiotic. Anaerobic phototrophic starter cultures (10 mL) were inoculated with the YPSMOPS grown cells (500 µL) in FW medium plus antibiotic and grown with hydrogen. After cultures grew to an OD_660_ ~ 1.2, the starter cultures were diluted 1:50 in 500 mL FW medium with antibiotic and grown photoautotrophically with hydrogen to an OD_660_ ~ 1.2. Cultures were harvested by centrifugation (10,000 x *g* for 30 min) and stored at -80^o^C. Cell pellets were thawed, fractionated, and PioA was purified using His affinity purification as described in the ‘Protein expression and purification from *E. coli’* section.

**Antibody production.** For the antibody against the N-terminus domain of PioA, the 200 aa-region (41-240 aa) was produced with an N-terminus GST-tag and purified using GST-affinity purification. The GST-N-terminus-PioA fusion protein was confirmed using the anti-GST antibody. The fusion protein (~500 µg) bands were cut out from the multiple Coomassie-stained SDS-PAGE gels and used for polyclonal antibody generation (Cocalico Bio. Inc.). Anti-peptide antibodies against PioA and a polyclonal antibody against PioB were generated by GenScript Inc. Two 14 aa long C-terminal peptides of PioA, ‘RNIKSGKVTPQGKM’ (294-307 aa) and ‘GGREKGGIRSFRPT’ (324-337 aa) were used for anti-peptide antibody production. A 169 aa C-terminus region of PioB (542-810aa) was synthesized (GenScript Inc.) and used for polyclonal antibody production.

**Heme Stains and Immunoblots.** Heme stains were performed as previously described (6). Protein samples were resolved by 12% (w/v) sodium dodecyl sulfate−polyacrylamide gel electrophoresis (SDS-PAGE) before heme staining or immunoblots. All membrane fractions of TIE-1 were heated to 90^o^C for 3 min before loading on to SDS-PAGE, unless noted otherwise in the text. In immunoblots, proteins were probed with the following antibodies: anti-PioA_N_ (1/2,000); anti-PioA_C_ (1/1,000); anti-PioB (1/500) and Protein A peroxidase (Sigma-Aldrich) was used a secondary label, and the Immobilon Western Chemiluminescent HRP Substrate (Millipore) was used for signal detection. Imaging was performed with LI-COR Odyssey Fc (LI-COR Biosciences). Near equal loading of total protein was assessed by Coomassie Blue staining. All the heme stain blots were washed with stripping buffer and confirmed for no signal for heme before they were used for immunoblots. Coomassie stained protein bands were identified by mass spectrometry at Proteomics and Mass Spectrometry facility, Donald Danforth Plant Science Center (DDPSC).

**RNA preparation and RT-qPCR.** *R. palustris* TIE-1 cultures were sampled in an anaerobic chamber and immediately mixed 1:1 with RNA*later* (Qiagen, USA) and incubated for 5 min at room temperature. The cells were harvested by centrifugation (10,000 x *g* for 5 min) and stored at -80^o^C. RNA was extracted using the RNeasy Mini Kit following the manufacturer’s recommendations (Qiagen, USA). Optizyme RNAase inhibitor (Thermofisher, USA) was added and DNA removal from the sample was performed using Turbo DNA-*free*™ Kit (Ambion, USA). PCR was performed to test the purity of RNA samples. cDNA was synthesized from the purified RNA using the iScript™ cDNA synthesis kit. The *pioA* expression analysis was performed using RT-qPCR with the comparative Ct method. Primers for RT-qPCR (Table S2) were designed using Primer3 v4.1.0 (http://primer3.ut.ee) using the programs default parameters. Primer efficiencies were measured according to the manufacturer's recommendations. *clpX* and *recA* were used as internal standards (7). The Bio-Rad iTaq™ Universal SYBR Green Supermix and the Bio-Rad CFX Connect™ Real-Time System Optics ModuleA machine (Bio-Rad Laboratories, Inc., Hercules, CA) were used for all quantitative assays following the manufacturer’s protocols.

**Microfluidic bioelectrochemical cell (µ-BEC) and conditions.** The µ-BECs were assembled and used as previously described (8). TIE-1 strains were grown photoautotrophically with hydrogen to OD_660_ ~1. The pregrown cultures were harvested, washed and concentrated to OD_660_ ~3 before injected into the µ-BEC using a FLOW EZ™ Fluigent Microflow Controller (Le Kremlin-Bicêtre, France) with 5 kPa of 80% N_2_ and 20% CO_2_ mixed gas. Bacterial cells were incubated in µ-BECs with working electrodes poised at +100 mV vs. SHE under illuminated conditions with a single 50 W halogen light bulb at a distance of 25 cm to establish biofilms. Once a stable current density under illuminated conditions (~-100 nA cm^-2^) was obtained, planktonic cells were washed out of the system with microfluidic control such that the attached biofilms account for the observed current densities. To monitor the light dependency of the process, a light “on-off” experiments were subsequently carried out at an interval of 10 seconds for a total of 900 seconds. Similarly, the experiments were also performed under continuous light for 900 seconds. The microfluidic flow was not applied during the electrochemical data collection.

**REFERENCES**

1. Ehrenreich A, Widdel F. 1994. Anaerobic oxidation of ferrous iron by purple bacteria, a new type of phototrophic metabolism. *Appl Environ Microbiol* **60**:4517-4526.
2. Jiao Y, Newman DK. 2007. The pio operon is essential for phototrophic Fe (II) oxidation in *Rhodopseudomonas palustris* TIE-1. *J Bacteriol* **189**:1765-1773.
3. Feissner RE, Richard-Fogal CL, Frawley ER, Loughman JA, Earley KW, Kranz RG. 2006. Recombinant cytochromes c biogenesis systems I and II and analysis of haem delivery pathways in *Escherichia coli*. *Mol Microbiol* **60**:563-577.
4. Sutherland MC, Rankin JA, Kranz RG. 2016. Heme trafficking and modifications during system I cytochrome c biogenesis: insights from heme redox potentials of Ccm proteins. *Biochemistry* **55**:3150-3156.
5. Egland PG, Gibson J, Harwood CS. 1995. Benzoate-coenzyme A ligase, encoded by badA, is one of three ligases able to catalyze benzoyl-coenzyme A formation during anaerobic growth of *Rhodopseudomonas palustris* on benzoate. *J Bacteriol* **177**:6545-6551.
6. Feissner R, Xiang Y, Kranz RG. 2003. Chemiluminescent-based methods to detect subpicomole levels of c-type cytochromes. *Anal Biochem* **315**:90-94.
7. Bose A, Gardel EJ, Vidoudez C, Parra EA, Girguis PR. 2014. Electron uptake by iron-oxidizing phototrophic bacteria. *Nat Commun* **5**:3391.
8. Guzman MS, Rengasamy K, Binkley MM, Jones C, Ranaivoarisoa TO, Singh R, Fike DA, Meacham JM, Bose A. 2019. Phototrophic extracellular electron uptake is linked to carbon dioxide fixation in the bacterium *Rhodopseudomonas palustris*. *Nat Commun* **10**:1355. 1.
9. Casadaban MJ, Cohen SN. 1980. Analysis of gene control signals by DNA fusion and cloning in *Escherichia coli*. *J Mol Biol* **138**:179-207.
10. Jiao Y, Kappler A, Croal LR, Newman DK. 2005. Isolation and characterization of a genetically tractable photoautotrophic Fe (II)-oxidizing bacterium, *Rhodopseudomonas palustris* strain TIE-1. *Appl Environ Microbiol* **71**:4487-4496.
11. Duchow E, Douglas H. 1949. *Rhodomicrobium vannielii*, a new photoheterotrophic bacterium. *J Bacteriol* **58**:409-416
12. Tushar L, Sasikala C, Ramana CV. 2014. Draft genome sequence of *Rhodomicrobium udaipurense* JA643T with special reference to hopanoid biosynthesis. *DNA Res* **21**:639-647.
13. Quandt J, Hynes MF. 1993. Versatile suicide vectors which allow direct selection for gene replacement in gram-negative bacteria. *Gene* **127**:15-21.
14. Katzke N, Arvani S, Bergmann R, Circolone F, Markert A, Svensson V, Jaeger KE, Heck A, Drepper T. 2010. A novel T7 RNA polymerase dependent expression system for high-level protein production in the phototrophic bacterium *Rhodobacter capsulatus*. *Protein Expr Purif* **69**:137-146.
15. Bose A, Newman DK. 2011. Regulation of the phototrophic iron oxidation (pio) genes in *Rhodopseudomonas palustris* TIE‐1 is mediated by the global regulator, FixK. *Mol Microbiol* **79**:63-75.
